# Long-term Care Insurance and Health and Perceived Satisfaction of Older Chinese: Comparisons Between Urban/Rural Areas, Chronic Conditions, and Their Intersectionality

**DOI:** 10.34172/ijhpm.2023.7938

**Published:** 2023-12-11

**Authors:** Yinkai Zhang, Yu-Chih Chen, Julia Shu-Huah Wang

**Affiliations:** ^1^Department of Social Work & Social Administration, The University of Hong Kong, Hong Kong SAR, China; ^2^Social Policy Institute, Washington University in St. Louis, St. Louis, MO, USA; ^3^Department of Social Work, National Taiwan University, Taipei, Taiwan

**Keywords:** : Long-Term Supports and Services, Self-Rated Health, Depression, Satisfaction, China Health and Retirement Longitudinal Study (CH

## Abstract

**Background:** Evidence of the impact of long-term care insurance (LTCI) on health and well-being has predominantly come from developed countries. China officially launched its city-level LTCI policy in 2016. Recent evidence in China has shown that having an LTCI program contributes to positive health. However, it is unclear whether such positive policy effects were attributed to policy announcement or implementation effects, and whether the policy effects vary by locality, chronic conditions, and their intersectionality. This study examines whether there are longitudinal health benefits for older Chinese who are participating in LTCI, particularly considering their city location (urban/rural), whether they have chronic conditions, and the intersectionality.

**Methods:** Following the Andersen Behavioral Model, health and satisfaction outcomes of 9253 adults aged 60+ years were extracted from the 2015 and 2018 waves of the China Health and Retirement Longitudinal Study (CHARLS). Individual data were linked to census socioeconomic data with city-level characteristics and LTCI policy variable. Multilevel lagged regression models investigated the impact of LTCI policy on health and satisfaction with health services, after controlling for baseline individual- and city-level covariates.

**Results:** Of 125 cities in the dataset, 21 (16.8%) had adopted LTCI. These city inhabitants had significantly better self-rated health and higher satisfaction relative to cities without LTCI policies when environmental- and personal-level characteristics were modeled. Health benefits of LTCI were stronger after policy announcement and were particularly observed among rural older adults and those with chronic conditions. Results also suggest that LTCI’s positive effects on satisfaction spill over to middle-aged adults.

**Conclusion:** Expanding coverage and eligibility to LTCI for all older Chinese could improve health and well-being.

## Background

**Key Messages**

**Implications for policy makers**

- Implementing a long-term care insurance policy (LTCI) could positively affect health and satisfaction of older adults; such effects can be attributed to policy announcement and implementation effects.
- There is a complex interplay between policy effect, locality, and chronic conditions. Policy effects are evident in rural older adults and those with chronic conditions.
- Expansion of LTCI policy should target physically- and mentally-vulnerable individuals with limited economic resources, living in deprived or remote areas, and balances provision, affordability, and sustainability.

**Implications for the public**

long-term care insurance (LTCI) policy plays a critical role in improving perceived health-related outcomes by reducing downstream need for expensive hospital and residential care services for older people with poor health. Expanding policy coverage to include vulnerable older adults, such as those with chronic conditions and living in rural areas, could not only shape LTCI policy to be more comprehensive and effective but also improve their health and well-being in later life. Their perceptions of the LTCI policy, personal health choices, and health service utilization can be important in shaping the effectiveness of LTCI policy formulation and its subsequent implementation.

Globally, the percentage of the population (aged 65+ years) is predicted to almost double, from 9% in 2019 to 16% in 2050.^1^ Aging is associated with frailty, chronic disease, and multimorbidity,^2^ often requiring long-term health and social support.^2,3^ Many countries have established policies to support long-term care (LTC) needs of older people (including associated costs).^4^ Long-term care insurance (LTCI) offers both in-kind services and cash benefits. Currently, LTCI comes in two forms (private market-oriented, or public mandated). Private LTCI includes eligibility and received benefits, and stresses the interplay between individuals’ choices and affordability, health conditions, personal responsibility, and market provision.^5^ Public LTCI is usually government-operated, with mandated participation, public financing, and basic provision of universal care for eligible citizens.^6^

 Although countries have implemented different concepts of LTCI, it generally includes home- and community-based services (HCBS); institutional care for people with chronic illness or disability^5^; financial reimbursement for services; and cash allowances for related LTC costs.^3^ Predominately, evidence of impact has come from correlational studies using cross-sectional data conducted in developed societies (eg, Japan, South Korea, and the United States), and the longitudinal effects of these policies could not be optimally identified. Evidence suggests that LTCI enhances care provision and health promotion, and is associated with reduced financial burdens^7^ and better health and quality of life.^8^ This is reflected by measures such as improved perceived health and satisfaction, alleviated disability, lower behavioral symptoms, lower prevalence of dementia,^4^ and even reduced mortality rates.^2^ These positive health outcomes suggest that implementing LTCI could enhance care management systems,^4^ improve coordination of formal, informal, and preventive services,^9^ and encourage individuals to transfer between hospitals and LTC facilities.^10^

 However, the longitudinal impacts of LTCI are relatively under-researched, particularly in China.^11^ A recent study^9^ using 2014 and 2018 waves of the Chinese Longitudinal Healthy Longevity Survey showed that the LTCI program was positively associated with several outcomes, including reduced likelihood of reporting unmet activity of daily living–related need for care, care expenditure, and improved self-rated health as well as lower one-year mortality. This research provides key initial evidence on the positive impact of LTCI on health. However, there are two important questions need further explorations. First, it is unclear whether the positive policy effects could be attributed to policy announcement or implementation. Second, whether such positive policy effect remains homogeneous across areas, people with health issues, and the intersectionality, are unclear. This study aims to advance the field and knowledge on the policy-health nexus by incorporating these important considerations to further differentiate the LTCI policy effect.

###  LTCI in the Chinese Context

 China is the largest developing country in the world, with the highest global percentage of people aged 60+ years (estimated 280 million [19.8%] of the total population in China in 2022). This is expected to reach 400 million (30% of the total Chinese population) in 2040.^3^ Tested policies need to be in place soon to ensure that China can meet increasing aged-care service needs.^12^ Under the Confucian philosophy of filial piety, Chinese families have traditionally played a central role in providing LTC for older adults.^13^ However, the massive rural-to-urban working migration over the past 30 years has left behind millions of low-income older adults in rural areas with no family support for their LTC needs.^13^

 The Chinese government initially implemented several city-based, small-scale models of public LTCI policies, such as nursing insurance in Qingdao, social health insurance in Shanghai, and a means-tested model in Nanjing.^7^ The rationale behind testing different LTC models is to identify models that can cater for city differences in household registration (ie, *hukou*), economic development, size of aging populations, and fiscal capacity.^14^ The impacts of these LTCI pilots largely showed reduced financial burdens for aged care^7^ and increased uptake of HCBS.^15^

 These findings led the Chinese government to launch state-sponsored LTCI plans in 2016 in 35 selected metropolitan areas^16^ (See Supplementary file 1, Table S1). LTCI policies have since been expanded to cover rural residents and more cities between 2016 to 2020.^14^ LTCI provision differs in each area in terms of reimbursement levels, eligibility, and service provision. LTCI policies are administered by the Human Resources and Social Security Bureau in each metropolitan area.^15^ As all LTCI pilots rely on China’s medical insurance funds, eligibility is similar to medical insurance that covers residents aged 16 years and older within respective *hukou*.^14^ This household registration system was established in 1958, where individuals are assigned an agricultural or non-agricultural (ie, urban) household registration status in a given location, based on their parents’ *hukou* status. The *hukou* system is a major contributor to social stratification in China. The *hukou* system governs population movement and defines individuals’ rights to social welfare (eg, medical insurance and pension) and services (eg, education, employment, healthcare) in urban and rural areas.^17^ Traditionally, individuals have limited opportunities to change *hukou* status.

 Contributions to LTCI vary, with respect to city-specific economic development, and demand for long-term aged-care services and supports. In general, the LTCI reimbursement ratio is capped at 70% of actual LTC cost,^15^ though this varies per city.^12^ Insurees can claim reimbursement for community and home care services (eg, daily activity care) and institutional nursing care (eg, social hospitalization), and some older people receive cash allowances.^14,15^ By the end of June 2019, 88.54 million insurees could access LTCI. Retired people, people who had not made prior contributions to LTCI, and people suffering long-term functional disabilities (six months or more), can receive LTCI benefits if they previously had medical insurance.^11^

###  Conceptual Framework

 The Andersen Behavioral Model^18^ postulates that healthcare systems, external environmental factors, and population characteristics, influence perceived health status and consumer satisfaction. Allocation of financial resources via policies such as LTCI to individuals, and agencies that provide care, may influence individuals’ behaviors in accessing care services, leading to changes in health status.^19^ However, policy affects health could be further attributed to the announcement and implementation effects.^20^ The former indicates that the anticipated benefits may affect health when individuals learn about future policy implementation. The latter suggests that services or programs established by the policy may shape individuals’ health when the policy has been executed.^21^ It is important to distinguish the health impacts from policy announcement and policy implementation to understand the mechanisms through which policy shapes health.

 External environmental factors represent contextual socioeconomic characteristics that can impact on health-related outcomes.^22^ These include gross domestic product (GDP),^23^ physical environment (urban or rural areas, or green spaces),^22^ low-income population rates,^24^ and access to healthcare and social services.^25^

 Population characteristics include predisposing, enabling, and needs-based factors.^18^ Predisposing factors include demographic and social structural attributes such as occupation and education, related to health. In the Chinese context, factors embedded in value and sociocultural systems (eg, family, marriage, education system) can be stressors or buffers for good health. Research suggests that health stressors for married people, and those with high education levels, can result in severe externalizing mental status behaviors (eg, alcohol and drug abuse), compared with females or older adults.^26^ However, females and those who were illiterate demonstrate worse internalizing health stressors (eg, depression; see Zeng et al^27^). Enabling factors for health are socioeconomic resources that facilitate good health behaviors and participation. Research has shown that income and wealth were significantly related to better health outcomes, where being employed was associated with positive health outcomes.^22^ Needs factors are demands for care (health conditions, comorbidities), and personal behaviors and practices.^28^ Lifestyle behaviors (smoking, drinking, and physical activity)^22^ and social and productive engagement such as social connection and offering care^29^ are determinants of health outcomes in later life. Lastly, health utilization, as manifested by medical expenditures and hospital utilization,^10^ is also related to health outcomes.

 The Andersen Behavioral Model recognizes individual- and environmental-level characteristics of health, where health is affected by healthcare systems, contextual socioeconomic variables, and individual resources, attributes, lifestyle choices and behaviors. Based on this framework, this study examines the direct influences and the relative importance of LTCI policy on health outcomes when considering environmental contextual factors, and individuals’ predisposing, enabling, and needs factors.

###  Variations and Intersection by Locality and Existing Chronic Conditions

 There is a substantial health divide between rural and urban areas in China, reflecting rapid urbanization and city-centric focus on policy implementations.^30^ Delays in implementing services and infrastructure have been reported in rural areas, which may compromise meeting the care needs of rural older adults, compared to their urban counterparts. Urban-rural differences in health may be further exacerbated by variable LTCI implementation, as the policy prioritizes urban residents (only 40% rural residents are covered by the scheme).^14^ The presence of chronic conditions may also influence how LTCI policy affects long-term health. Chronic conditions, including stroke, mental health issues, diabetes, Alzheimer’s disease, and arthritis, incur greater levels of multimorbidity and mortality.^28^ However, individuals with pre-existing conditions are often charged a higher premium or even denied access to market health insurance. Even if they are accepted by insurance schemes, they may have limited coverage, and care provision may focus more on cure or diagnosis than prevention or health maintenance. In contrast, public LTCI may have protective effects on health, particularly for vulnerable individuals (such as those with chronic conditions) as the policy aims for inclusivity and social effectiveness by offering basic care provision and longer-term services and supports.^11^

 Although the LTCI policy may positively affect rural older adults and those with chronic conditions, it is important to consider the intersectionality between locality and chronic conditions. For example, rural older adults with chronic conditions may particularly be at higher risk of facing health disadvantages, and their health status may respond differently when they experience policy intervention. As suggested by Holman and Walker,^31^ research that only focuses on one aspect of social characteristics without investigating intersectional social status would produce an incomplete understanding of how policy could redress health disparities, patterned by social hierarchy and attributes. However, there has been no systematic evaluation of how LTCI policy operates across different localities, chronic conditions, and their intersectionality, particularly in the Chinese context.

###  This Study

 This study contributes to the current literature in three ways. First, it examines whether LTCI policy in China affects perceived health and satisfaction outcomes. Second, it considers the differential impacts of policy announcement and implementation effects in an attempt to extend current evidence (eg, Lei et al^9^). Lastly, it explores the potentially-heterogeneous policy impacts on subgroups of older Chinese adults (in urban and rural areas, by chronic illness or not) and intersectionality (locality combined with chronic conditions).

## Methods

###  Data and Sample

 This study used nationally-representative individual panel survey data linked with administrative data. Individual-level data were drawn from the China Health and Retirement Longitudinal Study (CHARLS), which collects robust economic and health information from respondents aged 45 years and older.^32^ Beginning in 2011, the CHARLS has surveyed approximately 18 000 residents from 125 cities, with follow-up surveys conducted in 2013, 2015, and 2018. As China launched the LTCI pilot in 2016, comparative data came from the 2015 (baseline) and 2018 waves for both individual- and city-level data. The city-level data (See environmental characteristics in Table 1) were extracted from administrative data sources available in 2015, including government reports, open sources, and websites. We linked the city-level administrative data and individual-level CHARLS data together using the CHARLS city identifier.

**Table 1:** Descriptive Statistics and Bivariate Analyses across Locality for Baseline City-Level Variables

**Variables**	**All Sample** ** (N = 9253)**	**Locality**			**All Sample** **(N = 125)**	**Whether Have LTCI Policy**		
		**Urban ** **(n = 3654)**	**Rural ** **(n = 5599)**	***t*****/χ^2^**		**Yes ** **(n = 21)**	**No ** **(n = 104)**	***t*****/χ^2^**
Healthcare system								
Whether have LTCI policy	16.05%	20.09%	13.42%	*χ^2^ = *73.11***	16.80%			
Environmental characteristics								
Urban	39.49%							
GDP	10.62 (0.53)	10.82 (0.53)	10.49 (0.49)	*t *= –31.47***	10.66 (0.55)	10.98 (0.54)	10.60 (0.54)	*t *= –2.97**
Low-income population rate	6.14%	2.78%	8.37%	*t *= 46.16***	5.45%	2.76%	5.93%	*t *= 2.56*
Green spaces	13.31 (4.53)	13.27 (4.13)	13.34 (4.78)	*t *= 0.72	13.40 (4.50)	15.88 (4.98)	12.85 (4.22)	*t *= –2.88**
Number of healthcare clinics	7.20 (3.48)	6.36 (3.05)	7.76 (3.63)	*t *= 19.19***	7.12 (3.59)	6.26 (2.82)	7.30 (3.72)	*t *= 1.22

Abbreviations: LTCI, long-term care insurance; GDP, gross domestic product. *Note.* Terms: GDP (the log-transformed number of GDP per capita, CNY/year), Low-income population rate (the percentage of people receiving *Dibao*, %), and Green spaces (the number of square meters per person), Number of healthcare clinics (the number of hospitals and health centers per 10 000 people). Means are out of the parentheses, standard deviation are in the parentheses. * *P *≤.05; ** *P *≤.01; *** *P *≤.001.

 Following earlier research, we selected people aged 60 years and older at baseline^30^ and excluded proxy respondents (n = 690).^27^ As location and physical conditions may influence associations between policy and health outcomes, the study further stratified analyses by urban-rural,^33^ presence (or not) of chronic diseases,^28,34^ and their intersectionality. The final analytical sample was 9253 respondents (urban: n = 3654, rural: n = 5599; no chronic diseases: n = 1349, with chronic diseases: n = 7904; urban with chronic diseases: n = 3178, urban without chronic diseases: n = 476, rural with chronic diseases: n = 873, rural without chronic diseases: n = 4726), including 7940 respondents who were interviewed in both waves and 1313 who were not present at both waves.

###  Measurement

####  Outcomes

 Self-rated health (as an indicator of physical health), depressive symptoms (as an indicator of psychological distress), and perceived satisfaction (as an indicator of psychological well-being) variables were identified, in line with the Andersen Behavioral Model and encompassing multiple dimensions of health and well-being outcomes established in prior research.^35^ Self-rated health was measured with a single-item question: “*Would you say your health is very good, good, fair, poor or very poor?”* The score ranged from 1 (*very good*) to 5 (*very poor*). This measure has been validated and widely used to measure self-report health among Chinese older adults.^23,27^ We reverse-coded the measure so that a higher score indicates better self-rated health.

 Depressive symptoms were assessed by a 10-item, 4-point (from 0 to 3) short form of the Center for Epidemiologic Studies Depression scale (CESD-10). It has been validated in a Chinese sample, showing satisfactory psychometric properties.^36^ Two positive items (*happy* and *hopeful*) were coded reversely. The total score ranges from 0 to 30; a higher score indicates a higher level of depressive symptoms (*α* = 0.80).

 Perceived satisfaction with healthcare and services was measured with a single-item question: “*Are you satisfied with the quality, cost, and convenience of local home and community care and medical services?”* The score ranges from 1 (*very satisfied*) to 5 (*very dissatisfied*). We reverse-coded the measure so that a higher score indicates higher satisfaction with care systems. These outcomes were selected from both 2015 and 2018 CHARLS.

####  Predictor

 The primary predictor was whether the city had adopted the LTCI policy between 2015-2018 (1 = *yes*;0 = *no*). Among the 35 LTCI pilots, 21 cities were matched with the 125 cities surveyed in the CHARLS. Therefore, the policy effect was estimated by comparing these 21 cities with 104 cities that did not have LTCI. To further differentiate policy impact into the announcement and implementation effects, we collected information on policy announcement and implementation dates across cities, and based on respondents’ last interview date we constructed an additional 3-level LTCI policy coding (1 = *no LTCI* [n = 104; 83.95% of respondents]; 2 = announced but not implemented [n = 3; 2.26% of respondents]; 3 = implemented [n = 18; 13.79% of respondents]).

####  City-Level Covariates

 In line with the Andersen Behavioral Model, environmental variables included: locality (0 = *rural*, 1 = *urban*), GDP, low-income population rates (ie, defined as the percentage of the number of people receiving *Dibao*, a statutory low-income welfare program), green spaces (ie, defined as the number of square meters per person), and healthcare service numbers (defined as the number of hospitals and health centers per 10 000 people). Table S2 provides details.

####  Individual-Level Covariates

 Four types of individual-level predictors were considered (predisposing characteristics, enabling resources, need, and health behaviors). Predisposing characteristics included age (years); gender (0 = *female*, 1 = *male*); education level (0 = *below secondary school*, 1 = *above secondary school*); *hukou* (0 = *rural*, 1 = *urban*); marital status (1 = *married*, 2 = *partnered*, 3 = *not married/partnered*); and employment status (0 = *no*, 1 = *yes*). Enabling resources included income (ie, wage, government transfer, pension income, other income) and financial wealth, both were continuous and log-transformed. Needs included a binary variable (0 = *no*, 1 = *yes*) of whether respondents have chronic diseases,^28,34^ with respondents coded as 1 if they reported they have been diagnosed with any of the following diseases (arthritis or rheumatism, high blood pressure [eg, hypertension], stomach/digestive diseases [except for tumor or cancer], heart problems [eg, heart attack, coronary heart disease, angina, congestive heart failure, or other heart problems], dyslipidemia [ie, elevation of low density lipoprotein, triglycerides, and total cholesterol, or a low high density lipoprotein level], lung diseases [eg, chronic bronchitis, emphysema, excluding tumors, or cancer], diabetes or high blood sugar, kidney diseases [except for tumor or cancer], asthma, liver diseases [except fatty liver, tumors, and cancer], stroke, memory problems [eg, Alzheimer’s, brain atrophy, or Parkinson’s], cancer or malignant tumor [excluding minor skin cancers], emotional, nervous, or psychiatric problems), activities of daily living (ADL;*range*: 0-6), instrumental ADL (*range*: 0-5), and three health outcomes as lagged control variables (ie, self-rated health, depressive symptoms, and satisfaction with health services). Health Behavior included smoking (0 = *no*, 1 = *yes*), drinking alcohol (0 = *no*, 1 = *yes*), physical activity (0 = *no*, 1 = *yes*), caregiving (0 = *no*, 1 = *yes*), and social engagement (0 = *no*, 1 = *yes*) if the respondents were engaged in activities such as interaction with friends, playing chess or going to community club, going to entertainment club, participating in a community-related organization, volunteering, and attending an educational or training course. Use of health services included log-transformed total expenditure of hospitalization and length of hospital stay.^10^ All individual-level variables were selected from 2015 wave of the CHARLS.

###  Statistical Analysis

 Descriptive and bivariate analyses (*t* tests and chi-square analyses) were conducted to describe and test differences in individual-level and city-level factors by urban-rural location and chronic condition. The intraclass correlation coefficient for self-rated health, depressive symptoms, and satisfaction with health services were 0.04, 0.07, and 0.06 (these results were consistent with models with zero intercepts), respectively, indicating that a moderate amount of variability^37^ was due to inter-city differences. Following earlier research,^38^ we used the linear multilevel regression on self-rated health, depressive symptoms, and satisfaction of health services. The multilevel models for each outcome were built in sequence: the base model only included LTCI policy. City-level contextual variables were added next, followed by individual-level predictors. Based on previous approaches, data were further stratified by urban-rural,^33^ chronic conditions,^28,34^ and their intersectionality (urban-chronic conditions, urban-no condition, rural-chronic conditions, and rural-no condition). Additional analyses of policy announcement and implementation effects were conducted to further differentiate the policy impacts on health. However, stratified analyses (locality, chronic conditions, and intersectionality) were not applied to the announcement and implementation policy effects to optimize statistical power. To account for time order, the 2018 outcomes were regressed on 2015 baseline individual- and city-level factors, with additional three baseline outcomes as lagged covariates. This approach has been widely used to examine policy effects on health outcomes.^38,39^ Maximum likelihood estimation was used for all models to ensure comparability. The model fits were evaluated using the Akaike information criterion (AIC) and Bayesian information criterion (BIC). Sample attrition approximated 14%, with participants lost from the sample being older and less healthy than those remaining (See Supplementary file 2, Table S3). To address missing values (the missing rate ranging from approximately 0.05% to 50.98% across predictors), age and important health variables were modeled as key covariates, and we created 20 imputed data sets using multiple imputations with chained equations.^40^ As the sensitivity tests (See Supplementary file 2, Table S4) showed that the model fits did not significantly improve when allowing slopes of individual predictors to be varied across city levels, we adopted random-intercept models to ensure statistical parsimony.^37^ Analyses were conducted using Stata 14.0.

## Results

###  Sample Characteristics

Table 1 reports the results of descriptive and bivariate analysis on city-level variables. Approximately one-third of respondents (39.49%) lived in urban areas. Of the 125 cities surveyed in the CHARLS, 21 had adopted LTCI policy (See Supplementary file 2, Table S5), reflecting 16.05% of the total sample. These cities had better economic development and more green spaces than the 104 non-LTCI cities. A clear urban-rural difference was observed in environmental characteristics, as urban areas had more LTCI recipients and better economic development (ie, higher GDP and lower poverty rates). Although rural areas had more healthcare clinics, these clinics may be underresourced and provide poor quality care due to inadequate support and funding, compared to urban areas.^33^ Over the years, the gap in health expenditures between rural and urban areas has continued to grow.^41^

Table 2 reports descriptive and bivariate analysis results on individual-level characteristics. The average age of participants was 68 years (SD = 6.60), and approximately three in four were married (76.45%). Gender was equally represented (males reflecting 49.63% sample) and 52.22% were employed. Although 85.43% participants had at least one chronic disease, they were relatively healthy, indicated by high ADL (M = 5.46) and IADL (M = 4.42) scores and with average scores in self-rated health, depressive symptoms, and satisfaction with health services. Approximately half the respondents reported drinking alcohol and smoking, 30%-40% of respondents engaged in physical (28.91%) and social activities (44.64%), and approximately one-tenth offered caregiving to others. The bivariate analyses showed that demographic characteristics, resources, need/health conditions, and health behavior varied significantly considering the localities with and without LTCI pilot, urban or rural areas, and the presence of chronic disease. This finding indicates that our controls on health outcomes at baseline and city-level indicators extraordinarily important to address the preexisting differences across LTCI pilot and non-pilot cities. We further conducted bivariate analyses to present the potential health differences by combining urban-rural status with and without the LTCI pilot (eg, urban-with LTCI, urban-no LTCI, rural-with LTCI, and rural-no LTCI), and the results showed significant health differences by the combination of locality and LTCI status (See Supplementary file 2, Table S6). These significant differences provided an empirical rationale for stratifying analyses by locality and chronic conditions.

**Table 2:** Baseline Descriptive Statistics and Bivariate Analyses across Locality and Chronic Disease for Baseline Individual-Level Variables

**Variables**	**All sample** **(N = 9253)**	**Whether Have LTCI Policy**			**Locality**			**Chronic Disease**		
		**Yes ** **(n = 1485)**	**No ** **(n = 7768)**	***t*****/χ^2^**	**Urban ** **(n = 3654)**	**Rural ** **(n = 5599)**	***t*****/χ^2^**	**One and Over ** **(n = 7904)**	**Without any ** **(n = 1349)**	***t*****/χ^2^**
**Population characteristics**										
Predisposing characteristics										
Age	68.04 (6.60)	67.91 (6.43)	68.06 (6.64)	*t *= 0.82	68.17 (6.68)	67.96 (6.55)	*t *= –1.50	68.13 (6.56)	67.54 (6.86)	*t *= –3.00**
Male	49.63%	49.63%	49.62%	χ^2^ = 0.01	48.23%	50.54%	*χ^2^ *= 4.75*	48.29%	57.45%	*χ^2^ *= 38.72***
Above secondary school	7.33%	8.36%	7.13%	χ^2^ = 2.77	13.92%	3.03%	*χ^2^ *= 386.21***	7.61%	5.71%	*χ^2^ *= 6.09*
Urban *hukou*	23.92%	28.67%	22.97%	χ^2^ = 21.00***	51.84%	5.74%	*χ^2^ *= 2400.00***	24.58%	20.19%	*χ^2^ *= 11.48***
Marital status										
Married	76.45%	79.67%	75.84%	χ^2^ = 10.14***	78.13%	75.35%	*χ^2^ *= 9.50**	76.28%	77.46%	*χ^2^ *= 0.90
Partnered	3.38%	1.89%	3.67%	χ^2^ = 12.14***	2.52%	3.95%	*χ^2^ *= 13.82***	3.40%	3.26%	*χ^2^ *= 0.07
Working	52.22%	51.75%	52.32%	χ^2^ = 0.16	35.16%	62.89%	*χ^2^ *= 655.31***	50.32%	63.11%	*χ^2^ *= 74.75***
Enabling resources										
Income	6.00 (3.56)	6.39 (3.71)	5.92 (3.53)	*t *= –3.84***	6.76 (3.94)	5.51 (3.21)	*t *= –13.97***	5.98 (3.58)	6.11 (3.45)	*t *= 0.99
Financial wealth	10.78 (2.08)	10.86 (2.11)	10.77 (2.07)	*t *= –1.20	11.47 (2.00)	10.37 (2.02)	*t *= –19.36***	10.79 (2.07)	10.76 (2.13)	*t *= –0.31
Need										
Baseline have chronic disease	85.43%	83.51%	85.79%	χ^2^ = 5.24*	86.98%	84.41%	*χ^2^ *= 11.69***			
Baseline self-rated health	2.09 (0.92)	2.20 (0.96)	2.07 (0.91)	*t *= –5.21***	2.18 (0.91)	2.04 (0.92)	*t *= –7.27***	2.00 (0.88)	2.61 (0.98)	*t *= 23.10***
Baseline depressive symptoms	8.38 (6.54)	7.71 (6.36)	8.51 (6.56)	*t *= 4.33***	7.17 (6.04)	9.17 (6.72)	*t *= 14.39***	8.76 (6.66)	6.19 (5.28)	*t *= –13.44***
Baseline satisfaction	3.37 (1.13)	3.55 (1.10)	3.34 (1.14)	*t *= –6.39***	3.24 (1.09)	3.46 (1.15)	*t *= 8.65***	3.35 (1.14)	3.54 (1.10)	*t *= 5.58***
Baseline ADL	5.46 (1.16)	5.55 (1.04)	5.44 (1.18)	*t *= –3.23**	5.58 (1.00)	5.38 (1.25)	*t *= –8.42***	5.40 (1.21)	5.80 (0.69)	*t *= 11.80***
Baseline instrumental ADL	4.42 (1.08)	4.51 (1.01)	4.41 (1.09)	*t *= –3.36***	4.57 (0.96)	4.33 (1.14)	*t *= –10.36***	4.39 (1.11)	4.65 (0.84)	*t *= 8.32***
**Health behavior**										
Personal health practices										
Drinking	47.01%	47.71%	46.87%	χ^2^ = 0.35	45.81%	47.78%	*χ^2^ *= 3.39	46.75%	48.48%	*χ^2^ *= 1.38
Smoking	46.85%	48.99%	46.44%	χ^2^ = 3.25	44.64%	48.28%	*χ^2^ *= 11.67***	45.91%	52.34%	*χ^2^ *= 19.13***
Physical activity	28.91%	26.47%	29.38%	χ^2^ = 2.53	17.96%	35.67%	*χ^2^ *= 163.37***	28.08%	33.53%	*χ^2^ *= 8.46**
Caregiving	11.70%	11.79%	11.68%	χ^2^ = 0.02	11.61%	11.76%	*χ^2^ *= 0.05	11.75%	11.42%	*χ^2^ *= 0.12
Social engagement	44.64%	45.21%	44.54%	χ^2^ = 0.23	51.56%	40.17%	*χ^2^ *= 114.89***	44.80%	43.76%	*χ^2^ *= 0.50
Use of health services										
Medical expenditures	1.40 (3.24)	1.38 (3.22)	1.41 (3.24)	*t *= 0.36	1.55 (3.43)	1.31 (3.10)	*t *= –3.56**	1.53 (3.35)	0.66 (2.31)	*t *= –9.13***
Hospital utilization	0.27 (0.74)	0.27 (0.76)	0.28 (0.74)	*t *= 0.47	0.28 (0.73)	0.26 (0.75)	*t *= –1.52	0.30 (0.77)	1.12 (0.50)	*t *= –8.41***
**Outcomes (in 2018)**										
Self-rated health	2.89 (1.01)	3.08 (1.06)	2.86 (0.99)	*t *= –7.01***	3.00 (0.99)	2.83 (1.01)	*t *= –7.12***	2.81 (0.99)	3.37 (1.00)	*t *= 17.65***
CESD-10	8.79 (6.62)	7.90 (6.41)	8.97 (6.65)	*t *= 5.10***	7.61 (6.29)	9.50 (6.72)	*t *= 11.91***	9.13 (6.68)	6.84 (5.93)	*t *= –10.66***
Satisfaction with health services	3.38 (1.13)	3.50 (1.14)	3.36 (1.12)	*t *= –4.23***	3.28 (1.07)	3.44 (1.15)	*t *= 6.20***	3.36 (1.13)	3.52 (1.09)	*t *= 4.59***

Abbreviations: LTCI, long-term care insurance; CESD-10, Center for Epidemiologic Studies Depression scale; ADL, activities of daily living. *Note.* Individual-level variables (N= 9253). Income, wealth, GDP, and medical expenditures were presented in natural logarithm. Means are out of the parentheses, standard deviations are in the parentheses. * *P *≤.05; ** *P *≤.01; *** *P *≤.001.

###  Multilevel Analyses

####  Overall Sample

Table 3 presents the multilevel models for three outcomes. LTCI policy was significantly associated with better subsequent self-rated health (*b* = 0.225, *P*< .001), higher satisfaction with health services (*b* = 0.143, *P*< .05), and lower depressive symptoms (*b* = −0.827, *P*< .05). However, the effect of LTCI policy on depressive symptoms was non-significant when controlling for environmental and individual characteristics. In contrast, LTCI policy had a robust association with self-rated health (*b* = 0.102, *P*< .001) and satisfaction with health services (*b* = 0.109, *P*< .05), although positive effects were attenuated after the inclusion of individual and city-level covariates. The model fits (ie, lower AIC and BIC) gradually improved when the city- and individual-level variables were serially added to the model.

**Table 3:** Multilevel Models of the Predictors of Health Outcomes among Chinese Older Adults (N = 9253)

	**Self-Rated Health**			**Depressive Symptoms**			**Satisfaction With Health Services**		
**Model**	**M1**	**M2**	**M3**	**M4**	**M5**	**M6**	**M7**	**M8**	**M9**
	***b***	***b***	***b***	***b***	***b***	***b***	***b***	***b***	***b***
**Healthcare system**^a^									
Whether have LTCI policy	0.225*** (0.050)	0.161*** (0.044)	0.102*** (0.032)	–0.827* (0.389)	–0.294 (0.319)	–0.154 (0.218)	0.143* (0.063)	0.176** (0.062)	0.109* (0.052)
**Environmental characteristics**^a^									
GDP		0.097** (0.038)	0.054 (0.028)		–1.090*** (0.259)	–0.377* (0.177)		–0.073 (0.051)	–0.071 (0.041)
Low-income population rate		–1.440*** (0.358)	–0.821** (0.278)		11.885*** (2.386)	5.547** (1.746)		–0.172 (0.399)	–0.169 (0.352)
Urban		0.057 (0.030)	0.024 (0.028)		–0.882*** (0.194)	–0.133 (0.173)		–0.143*** (0.034)	–0.040 (0.036)
Green spaces		–0.009* (0.004)	–0.009*** (0.003)		0.079** (0.028)	0.044* (0.019)		0.009 (0.006)	0.005 (0.005)
Number of healthcare clinics		–0.004 (0.006)	–0.001 (0.004)		0.023 (0.037)	0.017 (0.026)		0.014 (0.008)	0.012* (0.006)
**Population characteristics**^a^									
Predisposing characteristics									
Age			–0.002 (0.002)			–0.016 (0.013)			0.005* (0.003)
Male			0.014 (0.032)			–1.201*** (0.213)			–0.110** (0.038)
Above secondary school			0.003 (0.043)			–0.828** (0.273)			0.002 (0.053)
Urban *hukou*			0.034 (0.030)			–0.020 (0.204)			–0.167*** (0.040)
Marital status									
Married			–0.033 (0.028)			–0.117 (0.178)			0.004 (0.033)
Partnered			–0.018 (0.061)			0.035 (0.412)			–0.007 (0.075)
Working			0.089*** (0.028)			0.148 (0.162)			0.036 (0.032)
Enabling resources									
Income			–0.002 (0.004)			–0.032 (0.023)			–0.001 (0.005)
Financial wealth			0.001 (0.009)			–0.134* (0.054)			–0.008 (0.010)
Need									
Baseline chronic disease			–0.206*** (0.030)			0.575** (0.182)			–0.031 (0.036)
Baseline self-rated health			0.353*** (0.013)			–0.553*** (0.082)			0.093*** (0.015)
Baseline depressive symptoms			–0.019*** (0.002)			0.410*** (0.012)			–0.009*** (0.003)
Baseline satisfaction			0.042*** (0.009)			–0.221*** (0.059)			0.258*** (0.012)
Baseline ADL			0.071*** (0.012)			–0.226** (0.080)			0.023 (0.015)
Baseline instrumental ADL			0.032** (0.013)			–0.261** (0.086)			–0.016 (0.015)
**Health behavior**^b^									
Personal health practices									
Drinking			0.057* (0.024)			–0.113 (0.147)			0.015 (0.029)
Smoking			–0.025 (0.029)			0.148 (0.183)			–0.041 (0.038)
Physical activity			–0.032 (0.040)			0.588* (0.240)			–0.065 (0.045)
Caregiving			0.039 (0.031)			–0.249 (0.195)			0.028 (0.040)
Social engagement			–0.008 (0.022)			–0.192 (0.135)			–0.037 (0.026)
Use of health services									
Medical expenditures			–0.014** (0.006)			0.055 (0.036)			–0.017* (0.007)
Hospital utilization			–0.038 (0.024)			0.050 (0.166)			–0.063 (0.032)
Attrition			0.007 (0.037)			–0.040 (0.222)			0.022 (0.038)
Model statistics									
–2 Log likelihood	26 173.23	26 089.88	23 528.55	60 945.42	60 781.44	57 789.61	28 215.86	28 177.66	27 192.60
AIC	26 181.23	26 107.88	23 592.55	60 953.41	60 799.44	57 846.36	28 223.86	28 195.66	27 256.59
BIC	26 209.76	26 172.08	23 820.80	60 981.94	60 863.64	58 074.61	28 252.39	28 259.86	27 484.84

Abbreviations: M, model; LTCI, long-term care insurance; GDP, gross domestic product; ADL, activities of daily living; AIC, Akaike information criterion; BIC, Bayesian information criterion. *Note.* ^a^ City-level variables (n = 125). ^b^ Individual-level variables (N = 9253). Estimates were rounding to three decimal points as some estimates were small; results were combined using 20 imputed data sets. Income, wealth, GDP, and medical expenditures were log-transformed. * *P *≤ .05; ** *P *≤ .01; *** *P *≤ .001.

Table 4 reports effects of policy announcement and implementation. Although the main results in Table 3 showed that LTCI policy positively impacts self-rated health and satisfaction with health services, these health outcomes responded differently to policy announcement and implementation. Policy announcement and implementation were both related to better self-rated health, and further comparisons in margins analysis from Model 3a, 6a and 9a (See Supplementary file 2, Table S7 and Figure S1) suggested that policy announcement effects on self-rated health and satisfaction with health services were stronger than implementation effects. However, satisfaction with health services was significantly improved only by policy announcement. We also found that policy implementation could reduce depressive symptoms, but this finding became non-significant when controlling for covariates.

**Table 4:** Multilevel Analysis of the LTCI Policy Announcement and Implementation Time (N = 9253)

	**Self-Rated Health**			**Depressive Symptoms**			**Satisfaction With Health Services**		
**Model**	**M1a**	**M2a**	**M3a**	**M4a**	**M5a**	**M6a**	**M7a**	**M8a**	**M9a**
	***b***	***b***	***b***	***b***	***b***	***b***	***b***	***b***	***b***
LTCI status (ref: no LTCI)									
Announced	0.425*** (0.113)	0.429*** (0.099)	0.280*** (0.075)	–0.142 (0.815)	–0.503 (0.693)	–0.371 (0.502)	0.472*** (0.131)	0.440*** (0.130)	0.290** (0.113)
Implemented	0.179*** (0.055)	0.107* (0.047)	0.071* (0.033)	–1.018* (0.433)	–0.248 (0.345)	–0.117 (0.231)	0.058 (0.069)	0.109 (0.068)	0.070 (0.057)
LTCI status (ref: Announced)									
Implemented	–0.246* (0.125)	–0.322** (0.108)	–0.210** (0.081)	–0.876 (0.906)	0.255 (0.748)	0.255 (0.533)	–0.414** (0.146)	–0.331* (0.142)	–0.221 (0.123)

Abbreviations: M, model; LTCI, long-term care insurance. *Note.* All models were adjusted for the city- and individual-level variables. Standard errors are in the parentheses. * *P *≤ .05; ** *P *≤ .01; *** *P *≤ .001.

####  Subgroup Variations

**Figure 1:**
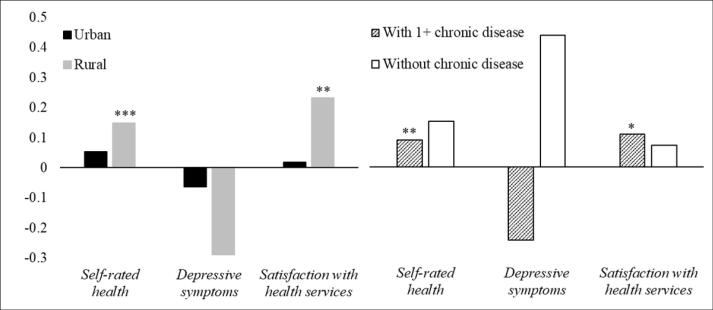
Effects of Long-term Care Insurance Policy on Health Outcomes, by Locality and Chronic Conditions. *Note.* All models were adjusted for the city- and individual-level variables in Table 2. The value of the vertical coordinate shows the coefficient of policy impacts. * *P *≤.05; ** *P *≤.01; *** *P *≤.001.

Figure 1 reports the effects of LTCI policy on health outcomes by location and chronic conditions, controlling for both city- and individual-level characteristics. Similar to the findings for the total sample, the LTCI policy was positively associated with self-rated health and satisfaction with health services, although policy effects operated differently across urban/rural areas and whether older adults had chronic conditions (See Supplementary file 2, Table S8). For rural older adults, LTCI policy was positively associated with better self-rated health (*b* = 0.150, *P*< .001) and satisfaction with health services (*b* = 0.223, *P*< .01). The positive association between LTCI policy and self-rated health (*b* = 0.091, *P*< .01) and satisfaction with health services (*b* = 0.111, *P*< .05) was also found among older adults with chronic conditions. However, LTCI policy did not have a positive effect on health outcomes among urban older adults, or for those without chronic conditions.

**Figure 2:**
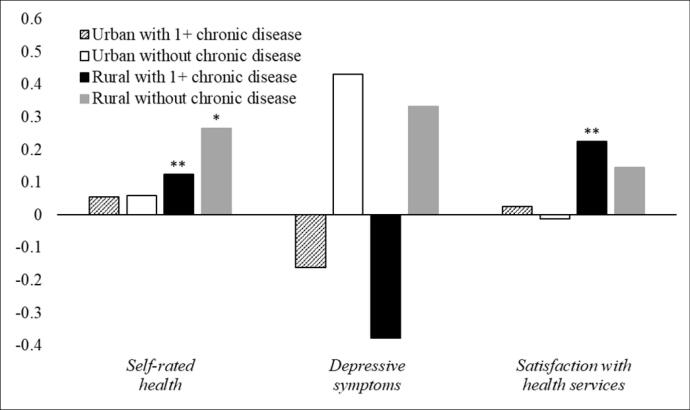
Intersectionality Analysis of Long-term Care Insurance Policy on Health Outcomes. *Note*. All models were adjusted for the city- and individual-level variables in Table 2. The value of the vertical coordinate shows the coefficient of policy impacts. * *P *≤.05; ** *P *≤.01; *** *P *≤.001.

Figure 2 presents the impacts of LTCI policy on intersectional subgroups. LTCI policy exerts a positive impact on self-rated health (*b* = 0.124, *P*< .01) and satisfaction with health services (*b* = 0.225, *P*< .01), particularly among rural older adults with chronic conditions. Furthermore, LTCI policy was positively associated with self-rated health (*b* = 0.267, *P*< .05) among rural older adults without chronic diseases. However, the LTCI policy effect was not related to depressive symptoms, and positive impacts were not observed among urban older adults regardless of chronic conditions (See Supplementary file 2, Table S9).

####  Robustness Check 

 We further conducted three sets of sensitivity tests to check the policy impacts. First, to validate the policy effects on older adults, we included a younger sample aged 45-59 as a comparison group that is presumed to be less affected by LTCI policies than older adults. The results (See Supplementary file 2, Table S10) showed that policy effects on self-rated health were only observed among older adults rather than middle-aged adults. Meanwhile, we observed evidence that the positive LTCI policy effects on older adults’ satisfaction with health services may also spill over to middle-aged adults. This can be due to middle-aged adults’ perception and anticipation of better LTC systems in their later life affecting their current assessment of health systems. Second, the binary measure of chronic conditions may not differentiate those with and without health conditions, as each chronic illness may have varied severity levels. An ideal approach is to construct a continuous functional comorbidity index^42^ to offer a nuanced investigation, but several key conditions (eg, osteoporosis, neurological disease, or degenerative disc disease) were not available in the CHARLS. Therefore, we followed Zhao et al^43^ to create a multimorbidity measure (defined as 2+ coexisting chronic conditions) to potentially address this issue and replace the chronic condition measure. The stratified results by multimorbidity (See Supplementary file 2, Table S11) were very similar to our original stratified analyses by chronic conditions, suggesting that the results of positive policy impacts, particularly among those with health conditions, were robust.

## Discussion

 This study demonstrates that adopting and implementing a policy to support LTC for older adults is critical in improving perceived health-related outcomes, especially for vulnerable individuals. Findings from the Chinese longitudinal data correspond with evidence from developed countries, and suggest that LTCI policy is positively associated long term with better self-rated health and perceived satisfaction. There is a complex interplay between policy effect, locality, and chronic conditions which must be considered in policy planning and implementation. Although cities with LTCI policy in place had better health and well-being outcomes in general, the LTCI could improve health and well-being for rural older adults and those with chronic conditions.

 The findings that LTCI policy was associated with better health and higher satisfaction are supported by a recent systematic review of mostly cross-sectional studies.^8^ Our study adds to the current evidence base (eg, Lei et al^9^) by differentiating policy impacts into announcement and implementation effects. While our analyses suggest that policy implementation was associated with improved self-rated health, policy announcement exerted stronger effects on positive self-rated health and satisfaction. This phenomenon, in line with Alpert,^44^ suggests that the effects of healthcare policy announcements tend to be stronger, primarily due to the anticipation of forthcoming health benefits that these announcements instill in individuals. The expected benefits from future policy implementation may change individuals’ perceived health and perception of current healthcare systems. Our findings are similar to a recent Colombian study^20^ where the anticipation effect may have a stronger impact on well-being than the implementation effect,^21^ especially in developing countries. Our findings suggest that studies on effects of LTCI on subjective outcomes (eg, Lei et al^9^) based on short-term follow up periods may in fact capture policy announcement effects rather than implementation effects. This demonstrates the importance of investigating longer-term follow-up data to adequately assess policy implementation effects.

 Additionally, how LTCI policy affects consumer satisfaction about the healthcare system could be framed by the Andersen Behavioral Model.^19^ For example, the LTCI policy supports allocation of resources to reduce the financial burden of community social care.^7^ This could encourage the use of HCBS by older adults and promote help-seeking behaviors, which in turn potentially decrease reliance on institutional- or hospital-based care.^15^ As a result, satisfaction with community service and healthcare could be improved by the affordability and availability of care due to LTCI policy implementation. However, the non-significant association between LTCI policy and depressive symptoms found in our study is inconsistent with prior research.^4,9^ Such inconsistency may be attributed to our longitudinal study design (compared with earlier cross-sectional studies from developed countries) or the potentially different ways that the LTCI policy had been implemented in participating Chinese cities.^2,4^ Current LTCI policy in China focuses more on physical functioning impairments rather than mental healthcare.^14^ This may be due to the strict assessment criteria for individuals with mental health issues. For example, in Guangzhou, people have to be clinically diagnosed with moderate or severe mental health problems to be eligible for LTCI benefits, and people with mild impairments are excluded.^11^ Therefore, the policy effect may be more evident in perceived general and physical health outcomes than depressive symptoms, especially in Chinese contexts.

 Our study confirms health differences between people living in rural and urban areas, and people with/without chronic conditions. Du et al^12^ noted that the effects on health of the LTCI policy may vary by residence registration (ie, *hukou*). Our sensitivity tests (See Supplementary file 2, Figure S2) stratified LTCI effect by *hukou* and found similar findings to stratification by locality, thus *hukou* was controlled as a covariate. Consistent with prior research on the health divide by locality and chronic illness,^28,30^ rural older adults and older people with chronic conditions have worse health than their urban or chronic illness-free counterparts. Such health gaps might be attributed to fewer available economic resources at individual level, and lagging development of health and social care infrastructure due to poorer community level financial systems,^33^ and higher multimorbidity and mortality risks associated with chronic disease.^2,28^ However, our study shows that LTCI policy was significantly associated with better self-rated health and higher satisfaction with the care system for those in rural areas and with chronic illness. The findings of the intersectionality models further support this, showing that positive policy impacts were evident among rural older adults regardless of whether they suffered from chronic conditions. As LTCI policy aims to reduce the financial burden of community care, such policy provision may improve access to previously unaffordable or unavailable, yet necessary, long-term support and services.^7^

 Our study has implications for Chinese aged care and health insurance policy design, as well as research. LTCI policy is clearly beneficial to individual health and well-being, especially for older adults living in rural areas and with chronic illnesses. This suggests that access to LTCI may reduce downstream need for expensive hospital and residential care for older people with poor health. Our results suggest that LTCI policy should be expanded to all Chinese cities, and should target not only all older adults, but also physically- and mentally-vulnerable individuals with limited economic resources, living in deprived or remote areas. Expansion of LTCI policy that balances eligibility, coverage, provision, affordability, and sustainability is recommended.^7,14^

 LTCI policy implementation is clearly an intervention that positively shapes health in participating Chinese cities. Reflecting this, in 2020, the Chinese government expanded LTCI to 14 more cities. When more recent CHARLS data is available, the impact of this policy expansion on health in these cities can be further explored. Our study highlights the need for more longitudinal research to test policy effects in different Chinese settings. Our use of the Andersen Behavioral Model^18^ enabled us to examine direct links between policy and health by accounting for city- and individual-level factors. However, we also found evidence of ‘thought’ mediators that require further investigation, such as people’s perceptions of the policy, personal health choices, and health service utilization. Our findings suggest the need for theory development, as the policy effect may operate differently in different localities, contexts and economies, and for different physical and mental conditions. Future studies can consider using analytic methods other than the multilevel models (eg, fixed-effect models, difference-in-difference models) to further test the robustness of policy effects. The utility of the Andersen Behavioral Model could be further assessed by testing how socio-structural characteristics, such as political and economic systems, welfare regimes, or institutional arrangements, affect the theoretical model assumptions. Lastly, using theoretical frameworks other than the Andersen Behavioral Model could be considered to explore further policy impacts. For example, how policy could affect individual or structural attributes in a feedback process^45^ could be useful in understanding the dynamics of the policy-health nexus.

 This study has limitations. First, policy information updates on cities’ administrative sources are not keeping pace with LTCI policy development. Although LTCI policy design features (eg, finance systems, reimbursement rates, or types of care provision) may differentially affect health,^4^ the extent to which LTCI policy implementation relates to policy design is yet understood, and this is further complicated by whether LTCI beneficiaries can accurately identify specific policy features in a local LTCI program. In our paper, we only measured LTCI policy in binary form (*yes/no*) as this binary form enabled parsimonious estimation of the average effect of LTCI implementation. We extend current literature by further exploring how timing of policy execution (ie, announced and implemented) affects health. Effects of detailed policy design features (eg, reimbursement levels, eligibility, service provisions, policy variations for urban or rural residents) warrant examination in future research studies. Second, the study window was restricted to 2015 and 2018 (dictated by the Chinese LTCI policy launch date in 2016, and the latest CHARLS survey wave in 2018). Future research should examine the prospective impacts of LTCI policy on health by further extending the post-policy implementation study period when data becomes available, allowing longer term follow-up to explore how the policy shapes health outcomes in later life. Third, CHARLS is a nationally-representative survey that begins at age 45 years, whereas our selected sample started at age 60 years. This could be a departure from national profiles.^32^ Fourth, the original design of the satisfaction measure in the questionnaire combines quality, cost, and convenience into a single question, which precludes further explorations on specific dimensions of satisfaction. Last, although the study measures were informed by the Andersen Behavioral Model, other important variables, such as health beliefs, combinations of particular disease variables (eg, comorbidity index), social and natural environment measures (eg, transportation, see Chao and Chen^46^), and barriers to LTCI access (eg, knowledge on LTCI, see Li and Jensen^5^) could not be examined as they were not available for all cities in CHARLS.

 Despite its limitations, this study contributes to current knowledge by providing longitudinal evidence of LTCI policy on the health and well-being of older Chinese adults. It demonstrates that policy effect is a complex interaction intertwined with locality and chronic conditions. Our study shows that irrespective of how and where it is implemented, LTCI policy promotes healthy ageing, particularly for rural older adults, and those with chronic conditions. This study supports the expansion of the coverage and eligibility of LTCI in China.

## Ethical issues

 This study used secondary data from CHARLS, the IRB approval number for the main household survey, including anthropometrics, is IRB00001052-11015.

## Competing interests

 Authors declare that they have no competing interests.

## Supplementary files

Supplementary file 1. Chinese LTCI Pilots Launched in 2016 and Baseline City Characteristics, Surveyed in CHARLS.

Supplementary file 2 contains Tables S3-S11 and Figures S1-S2.

## References

[1] United Nations. World Population Prospects 2019 Highlights. 2019. https://www.un.org/development/desa/publications/world-population-prospects-2019-highlights.html. Accessed June 17, 2019.

[2] Choi JK, Joung E (2016). The association between the utilization of long-term care services and mortality in elderly Koreans. Arch GerontolGeriatr.

[3] Wu B, Cohen MA, Cong Z, Kim K, Peng C (2021). Improving care for older adults in China: development of long-term care policy and system. Res Aging.

[4] Lee TW, Yim ES, Choi HS, Chung J (2019). Day care vs home care: effects on functional health outcomes among long-term care beneficiaries with dementia in Korea. Int J Geriatr Psychiatry.

[5] Li Y, Jensen GA (2012). Why do people let their long-term care insurance lapse? Evidence from the health and retirement study. Appl Econ Perspect Policy.

[6] Kim H, Kwon S (2021). A decade of public long-term care insurance in South Korea: policy lessons for aging countries. Health Policy.

[7] Yang W, Jingwei He A, Fang L, Mossialos E (2016). Financing institutional long-term care for the elderly in China: a policy evaluation of new models. Health Policy Plan.

[8] Chen L, Xu X (2020). Effect evaluation of the long-term care insurance (LTCI) system on the health care of the elderly: a review. J MultidiscipHealthc.

[9] Lei X, Bai C, Hong J, Liu H (2022). Long-term care insurance and the well-being of older adults and their families: evidence from China. Soc Sci Med.

[10] Feng J, Wang Z, Yu Y (2020). Does long-term care insurance reduce hospital utilization and medical expenditures? Evidence from China. Soc Sci Med.

[11] Wu J, Chen S, Wen H, Yi Y, Liao X (2020). Health status, care needs, and assessment for beneficiaries with or without dementia in a public long-term care insurance pilot in Guangzhou, China. BMC Health Serv Res.

[12] Du P, Dong T, Ji J (2021). Current status of the long-term care security system for older adults in China. Res Aging.

[13] Chui EW-T (2012). Caring for our seniors – private issue or public? The Asian scene. J Asian Public Policy.

[14] Zhu Y, Österle A (2019). China’s policy experimentation on long-term care insurance: implications for access. Int J Health Plann Manage.

[15] Zhang Y, Yu X (2019). Evaluation of long-term care insurance policy in Chinese pilot cities. Int J Environ Res Public Health.

[16] National Healthcare Security Administration. Guidance on the Expansion of the Long-Term Care Insurance System Pilot [Chinese]. 2020. http://www.nhsa.gov.cn/module/download/downfile.jsp?classid=0&filename=b9f5c190706640ef8b452b3cf959e5bc.pdf. Accessed September 16, 2020.

[17] Cai F (2011). Hukou system reform and unification of rural–urban social welfare. China World Econ.

[18] Andersen RM (1995). Revisiting the behavioral model and access to medical care: does it matter?. J Health Soc Behav.

[19] von Lengerke T, Gohl D, Babitsch B. Re-revisiting the Behavioral Model of Health Care Utilization by Andersen: a review on theoretical advances and perspectives. In: Janssen C, Swart E, von Lengerke T, eds. Health Care Utilization in Germany. New York, NY: Springer; 2014:11-28. 10.1007/978-1-4614-9191-0_2.

[20] Ladino JF, Saavedra S, Wiesner D (2021). One step ahead of the law: the net effect of anticipation and implementation of Colombia’s illegal crops substitution program. J Public Econ.

[21] Wang JSH, Kaushal N (2019). Health and mental health effects of local immigration enforcement. Int Migr Rev.

[22] Salgado M, Madureira J, Mendes AS, Torres A, Teixeira JP, Oliveira MD (2020). Environmental determinants of population health in urban settings A systematic review. BMC Public Health.

[23] Sun R, Gu D (2008). Air pollution, economic development of communities, and health status among the elderly in urban China. Am J Epidemiol.

[24] Hsu HC, Bai CH (2021). Social and built environments related to cognitive function of older adults: a multi-level analysis study in Taiwan. Int J Environ Res Public Health.

[25] Park S, Cho J, Chen YC (2019). Subsidized housing and geographic accessibility to neighborhood resources for low-income older people: from later year social exclusion perspective. Geoforum.

[26] Chiavegatto Filho ADP, Sampson L, Martins SS (2017). Neighbourhood characteristics and mental disorders in three Chinese cities: multilevel models from the World Mental Health Surveys. BMJ Open.

[27] Zeng Y, Chen YC, Lum TYS (2021). Longitudinal impacts of grandparent caregiving on cognitive, mental, and physical health in China. Aging Ment Health.

[28] Shu Z, Han Y, Xiao J, Li J (2019). Effect of medical insurance and family financial risk on healthcare utilisation by patients with chronic diseases in China: a cross-sectional study. BMJ Open.

[29] Chao SF, Chen YC (2019). Environment patterns and mental health of older adults in long-term care facilities: the role of activity profiles. Aging Ment Health.

[30] Wang Y, Gonzales E, Morrow-Howell N (2017). Applying WHO’s age-friendly communities framework to a national survey in China. J Gerontol Soc Work.

[31] Holman D, Walker A (2021). Understanding unequal ageing: towards a synthesis of intersectionality and life course analyses. Eur J Ageing.

[32] Zhao Y, Hu Y, Smith JP, Strauss J, Yang G (2014). Cohort profile: the China Health and Retirement Longitudinal Study (CHARLS). Int J Epidemiol.

[33] Reijneveld SA (2002). Neighbourhood socioeconomic context and self reported health and smoking: a secondary analysis of data on seven cities. J Epidemiol Community Health.

[34] Cao L, Gao J, Xia Y (2021). The effects of household solid fuel use on self-reported and performance-based physical functioning in middle-aged and older Chinese populations: a cross-sectional study. Ecotoxicol Environ Saf.

[35] Nakamura JS, Hong JH, Smith J (2022). Associations between satisfaction with aging and health and well-being outcomes among older US adults. JAMA Netw Open.

[36] Chen H, Mui AC (2014). Factorial validity of the Center for Epidemiologic Studies Depression Scale short form in older population in China. Int Psychogeriatr.

[37] Luke DA. Multilevel Modeling. Thousand Oaks, CA: SAGE Publications; 2004. 10.4135/9781412985147.

[38] Martinussen PE, Rydland HT (2021). Is a decentralised health policy associated with better self-rated health and health services evaluation? A comparative study of European countries. Int J Health Policy Manag.

[39] Gruneir A, Miller SC, Intrator O, Mor V (2007). Hospitalization of nursing home residents with cognitive impairments: the influence of organizational features and state policies. Gerontologist.

[40] White IR, Royston P, Wood AM (2011). Multiple imputation using chained equations: issues and guidance for practice. Stat Med.

[41] Chen Y, Yin Z, Xie Q (2014). Suggestions to ameliorate the inequity in urban/rural allocation of healthcare resources in China. Int J Equity Health.

[42] Groll DL, To T, Bombardier C, Wright JG (2005). The development of a comorbidity index with physical function as the outcome. J Clin Epidemiol.

[43] Zhao Y, Atun R, Oldenburg B (2020). Physical multimorbidity, health service use, and catastrophic health expenditure by socioeconomic groups in China: an analysis of population-based panel data. Lancet Glob Health.

[44] Alpert A (2016). The anticipatory effects of Medicare Part D on drug utilization. J Health Econ.

[45] Goss KA (2010). Civil society and civic engagement: towards a multi-level theory of policy feedbacks. J Civ Soc.

[46] Chao SF, Chen YC (2019). Environment patterns and mental health of older adults in long-term care facilities: the role of activity profiles. Aging Ment Health.

