# Supplementary material for: Long-term Care Insurance and Health and Perceived Satisfaction of Older Chinese: Comparisons Between Urban/Rural Areas, Chronic Conditions, and Their Intersectionality
Source: Int J Health Policy Manag. 2023 Dec 11;12:7938. doi: 10.34172/ijhpm.2023.7938 (PMC10843175; doi:10.34172/ijhpm.2023.7938)
Supplement: Supplementary file 1 — Chinese LTCI Pilots Launched in 2016 and Baseline City Characteristics, Surveyed in CHARLS. [file ijhpm-12-7938-s001.pdf]

Article title: Long-term Care Insurance and Health and Perceived Satisfaction of Older Chinese: Comparisons Between Urban/Rural Areas, Chronic Conditions, and Their Intersectionality

Journal name: International Journal of Health Policy and Management (IJHPM)

Authors' information: Yinkai Zhang[1], Yu-Chih Chen[1,2]*, Julia Shu-Huah Wang[3]

[1]Department of Social Work & Social Administration, The University of Hong Kong, Hong Kong SAR, China.

[2]Social Policy Institute, Washington University in St. Louis, St. Louis, MO, USA.

[3]Department of Social Work, National Taiwan University, Taipei, Taiwan.

***Correspondence to:** Yu-Chih Chen; Email: yuchih@hku.hk

Citation: Zhang Y, Chen YC, Wang JSH. Long-term care insurance and health and perceived satisfaction of older Chinese: comparisons between urban/rural areas, chronic conditions, and their intersectionality. Int J Health Policy Manag. 2023;12:7938. doi:10.34172/ijhpm.2023.7938

Supplementary file 1. Chinese LTCI Pilots Launched in 2016 and Baseline City Characteristics, Surveyed in CHARLS

Table S1. The Chinese LTCI Pilots Launched in 2016, Surveyed in CHARLS.

City ID	City	Reimbursement proportion of actual expense	Maximum amount[1]	Cash allowance[1]	Who is eligible? Employees	Who is eligible? Non-working residents	Source
13	Chengdu	75% (home care)<br>70% (institutional care)	N/A	Not offered	Yes	No	http://gk.chengdu.gov.cn/govInfoPub/detail.action?id=88855&tn=6
25	Chengde	N/A	50 (institutional aged care)<br>60 (institutional medical care)	Not offered	Yes	No	http://hbrb.hebnews.cn/pc/paper/c/201708/17/c14732.html
29	Shangrao	N/A	30 (home care)<br>40 (institutional care)	15	Yes	No	http://www.zgsr.gov.cn/zgsr/zcwjz/201908/4f8d4890c7484313a9b166b9b34c62ea.shtml
40	Suzhou	N/A	25 (home care for moderately disabled people)<br>30 (home care for severely disabled people)<br>20 (institutional care for moderately disabled people)<br>26 (institutional care for severely disabled people)	Not offered	Yes	Yes	http://ybj.suzhou.gov.cn/szybj/chxi/202008/c73ab150aea74364a0030e9aecf76558.shtml
47	Chongqing	N/A	50 (home care and institutional care)	Not offered	Yes	No	http://rlsbj.cq.gov.cn/zwxx_182/tzgg/201712/t20171222_6719581_wap.html
53	Qiqihaer	50% (home care)<br>55% (institutional aged care)<br>60% (institutional medical care)	20 (home care)<br>25 (institutional aged care)<br>30 (institutional medical care)	Not offered	Yes	No	http://m.law-lib.com/law/law_view.asp?id=585249
59	Guangzhou	90% (home care)<br>75% (institutional care)	103.5 (home care)<br>90 (institutional care)<br>33.33 (hospital nursing care)	Not offered	Yes	No	http://www.gz.gov.cn/zwfw/zxfw/sbfw/content/post_2857250.html
72	Shanghai	90% (home care)<br>85% (institutional care)	N/A	Not offered	Yes	Yes	https://www.shanghai.gov.cn/nw41430/20200823/0001-41430_54809.html
75	Ningbo	N/A	40 (institutional care)	Not offered	Yes	No	http://www.ningbo.gov.cn/art/2018/5/17/art_1229541874_59034707.html
81	Jilin	70% (institutional care for non-working residents)<br>80% (institutional care for employees)	N/A	Not offered	Yes	Yes	http://www.jlcity.gov.cn/zt/zwzt/hqhmzc/shmszc/201912/t20191210_690818.html
89	Anqing	50% (institutional care)<br>60% (hospital nursing care)	25 (home care)<br>40 (institutional care)<br>50 (hospital nursing care)	Not offered	Yes	No	http://aqxxgk.anqing.gov.cn/show.php?id=505293

City ID	City	Reimbursement proportion of actual expense	Maximum amount[1]	Cash allowance[1]	Who is eligible?		Source
					Employees	Non-working residents	
96	Jingmen	80% (full-time home care)<br>100% (part-time home care)<br>75% (institutional care)<br>70% (hospital nursing care)	40 (part-time home care)<br>100 (full-time home care)<br>100 (institutional care)<br>150 (hospital nursing care)	Not offered	Yes	Yes	http://rlzyb.jcut.edu.cn/info/1008/1256.htm
101	Binzhou	N/A	45 (home care)<br>70 (institutional care, ADL<=20)<br>60 (institutional care, 20<ADL<=40)<br>50 (institutional care, 41<=ADL<=50)<br>260 (level 1 hospital nursing care for tracheotomy patients)<br>150 (level 1 hospital nursing care for other patients)<br>280 (level 2 hospital nursing care for tracheotomy patients)<br>180 (level 2 hospital nursing care for other patients)<br>300 (level 3 hospital nursing care for tracheotomy patients)<br>210 (level 3 hospital nursing care for other patients)	Not offered	Yes	No	http://m.law-lib.com/law/law_view.asp?id=611687
102	Qingdao	70% (first-rate paying non-working residents)<br>80% (second-rate paying non-working residents)<br>90% (employees)	N/A	Not offered	Yes	Yes	https://www.yanglaocn.com/shtml/20180320/1521532406114318.html
103	Jinan	N/A	50 (home care)<br>60 (institutional care)<br>200 (hospital nursing care)	Not offered	Yes	No	http://law.51labour.com/lawshow-98520.html
104	Zaozhuang	75% (home care)<br>75% (institutional care)	N/A	Not offered	Yes	No	http://zaozhuang.dzwww.com/news/zznews/201807/t20180703_16551818.html
105	Linyi	90% (home care)<br>85% (institutional care)<br>85%(level 1 hospital nursing care)<br>80% (level 2 hospital nursing care)<br>75% (level 3 hospital nursing care)	N/A	Not offered	Yes	No	https://www.sohu.com/a/195152981_100025093

City ID	City	Reimbursement proportion of actual expense	Maximum amount[1]	Cash allowance[1]	Who is eligible?		Source
					Employees	Non-working residents	
106	Liaocheng	75%	N/A	Not offered	Yes	No	http://liaocheng.iqilu.com/lcyaowen/2017/0822/3659629.shtml
107	Weihai	N/A	30 (partial home or institutional care)<br>40 (complete home or institutional care)<br>40 (complete institutional care)<br>200 (hospital nursing care)	Not offered	Yes	No	http://www.weihai.gov.cn/art/2018/4/12/art_51907_2101619.html
108	Weifang	N/A	60 (home or institutional care)<br>120 (level 1 hospital nursing care)<br>170 (level 2 hospital nursing care)<br>200 (level 3 hospital nursing care)	Not offered	Yes	No	https://www.sohu.com/a/213975675_607151
109	Dezhou	85% (institutional care)<br>80% (level 2 hospital nursing care)<br>75% (level 3 hospital nursing care)	30 (home care)<br>60 (institutional care)<br>120 (level 2 hospital nursing care)<br>180 (level 3 hospital nursing care)	Not offered	Yes	No	https://www.sohu.com/a/292260642_170177

Note:

[1] CNY/person/day.

Table S2. Baseline City Characteristics among 125 Cities in CHARLS.

City ID	City	GDP per capita[1]	Population[2]		Population received *Dibao*[2]		Low-income population rate[3]		Health services		Green spaces[5]	Source
			Urban	Rural	Urban	Rural	Urban	Rural	Total	Access to health care[4]		
1	Baoshan	21385.5	175.51	82.59	4.20	26.00	0.02	0.31	1337	5.18	6.98	http://www.tjcn.org/tjgb/25yn/32924_2.html
2	Chuxiong	27942.0	110.50	162.80	9.89	18.82	0.09	0.12	1720	6.29	12.11	http://www.tjcn.org/tjgb/25yn/32794.html
3	Zhaotong	13112.0	158.56	384.44	15.61	62.07	0.10	0.16	1980	3.65	7.02	http://www.tjcn.org/tjgb/25yn/32792.html
4	Kunming	59686.0	467.70	200.00	9.93	16.52	0.02	0.08	4490	6.72	10.38	http://www.km.gov.cn/c/2016-07-21/3410538.shtml
5	Lincang	20077.0	92.48	158.42	6.97	39.15	0.08	0.25	1380	5.50	11.24	http://www.lincang.gov.cn/lcszf-ydb/zjlc63/gmjjyshfzgk25/9864/index.html
6	Lijiang	22702.0	82.40	45.60	3.67	14.59	0.04	0.32	200	1.56	18.92	http://www.tjcn.org/tjgb/25yn/32793_5.html
7	Zhangzhou	55571.0	274.00	226.00	2.79	12.11	0.01	0.05	4479	8.96	14.53	http://www.zhangzhou.gov.cn/cms/html/zzsrmzf/2016-03-23/1584333641.html
8	Putian	57873.0	162.40	124.60	0.70	7.93	0.00	0.06	1321	4.60	12.67	http://www.putian.gov.cn/zwgk/tjxx_222/tjgb/201611/t20161108_500634.htm
9	Fuzhou	75259.0	507.75	242.25	1.40	7.75	0.00	0.03	4133	5.51	13.52	http://tjj.fuzhou.gov.cn/zz/zwgk/tjzl/ndbg/201603/t20160331_46755.htm
10	Ningde	52016.0	153.80	133.20	1.58	11.99	0.01	0.09	2978	10.38	15.49	http://www.tjcn.org/tjgb/13fj/32693_7.html
11	Haidong	26531.2	49.44	95.99	3.85	11.61	0.08	0.12	2060	14.16	5.81	http://www.tjcn.org/tjgb/29qh/32955.html

City ID	City	GDP per capita[1]	Population[2]		Population received *Dibao*[2]		Low-income population rate[3]		Health services		Green spaces[5]	Source
			Urban	Rural	Urban	Rural	Urban	Rural	Total	Access to health care[4]		
12	Guangan	31046.0	324.70	203.80	8.80	19.04	0.03	0.09	3500	6.62	20.14	http://www.guang-an.gov.cn/gasrmzfw/sjtjj/2017-11/08/content_bd5fb014cd9c434393f8869d4fdc8d19.shtml
13	Chengdu	74273.0	1047.61	418.19	3.40	10.90	0.00	0.03	8481	5.79	14.59	http://www.tjcn.org/tjgb/23sc/32950.html
14	Liangshan	28276.0	N/A	N/A	7.36	57.12	0.13	0.13	1088	2.13	N/A	http://tjj.lsz.gov.cn/sjfb/lstjgb/201606/t20160630_1176627.html
15	Nanchong	23881.0	278.74	357.66	22.88	54.72	0.08	0.15	8712	13.69	10.60	http://www.nanchong.gov.cn/news/show/642cb29f-83af-4cc0-beab-68f039918597.html
16	Yibin	34060.0	202.50	246.50	6.24	15.39	0.03	0.06	4963	11.05	9.25	http://tjj.yibin.gov.cn/sjfb/tjgb/201604/t20160411_612597.html
17	Ziyang	35595.4	141.00	215.90	3.98	16.55	0.03	0.08	4902	13.73	12.22	http://www.tjcn.org/tjgb/23sc/32783.html
18	Neijiang	32080.0	170.57	203.40	6.00	11.29	0.04	0.06	3195	8.54	9.26	http://tjj.neijiang.gov.cn/2016/07/1203790.html
19	Ganzi	18423.0	32.69	83.80	2.80	25.61	0.09	0.31	2725	23.39	1.44	http://www.gzz.gov.cn/gzzrmzf/c100048/201603/d05f7f24392a40bcbe745633f68181c1.shtml
20	Mianyang	35632.1	229.05	248.14	10.16	16.77	0.04	0.07	4417	9.26	11.02	http://www.tjcn.org/tjgb/23sc/32778_3.html
21	Meishan	34379.0	125.66	174.47	5.75	13.95	0.05	0.08	2057	6.85	11.37	http://www.tjcn.org/tjgb/23sc/32781.html
22	Baoding	29067.0	482.80	552.10	5.20	24.60	0.01	0.04	10017	9.68	9.86	http://www.tjcn.org/tjgb/03hb/34803_4.html
23	Shijiazhuang	50839.1	631.72	438.44	2.76	15.45	0.00	0.04	6656	6.22	15.57	http://www.sjz.gov.cn/col/1596018184396/2016/04/11/1598877483849.html
24	Cangzhou	41846.6	376.05	398.35	N/A	N/A	N/A	N/A	9299	12.01	10.38	http://www.tjcn.org/tjgb/03hb/32932.html
25	Chengde	38486.2	165.21	187.80	6.40	23.80	0.04	0.13	3732	10.57	24.57	http://www.tjcn.org/tjgb/03hb/32829_3.html
26	Jingdezhen	47062.1	104.22	59.83	N/A	N/A	N/A	N/A	1074	6.55	15.18	http://www.jdz.gov.cn/sjkf/tjgb/t284248.shtml
27	Nanchang	75879.0	379.48	150.81	5.65	9.72	0.01	0.06	2118	3.99	11.80	http://www.nc.gov.cn/ncszf/tjgb/201604/VU2E1OI7UOYIF0B4JIGX66MTLLHU4YLJ.shtml
28	Yichun	29459.0	247.05	304.15	N/A	N/A	N/A	N/A	N/A	0.00	15.28	http://tjj.yichun.gov.cn/news-show-553854.html
29	Shangrao	24633.0	317.89	353.61	N/A	N/A	N/A	N/A	8426	12.55	14.82	http://www.tjcn.org/tjgb/14jx/32911_6.html
30	Jiujiang	39505.0	243.99	238.59	7.79	17.02	0.03	0.07	2721	5.64	17.77	https://www.jiujiang.gov.cn/xxgk/xzwgk/jggk/tjxx/tjgb/201604/t20160408_1574106.html
31	Jian	27168.0	226.18	263.71	8.92	N/A	N/A	N/A	4781	9.76	17.09	http://www.tjcn.org/tjgb/14jx/32858_2.html
32	Ganzhou	27093.0	228.24	732.39	8.44	33.10	0.04	0.05	8839	9.20	10.37	http://www.tjcn.org/tjgb/14jx/32698_6.html
33	Akesu	47406.3	83.04	170.07	N/A	N/A	N/A	N/A	1532	6.05	N/A	http://www.tjcn.org/tjgb/31xj/32929_3.html
34	Beijing	106284.0	1877.70	292.80	8.50	4.90	0.00	0.02	10423	4.80	16.00	http://cn.chinagate.cn/reports/2016-07/18/content_38904966.htm
35	Xilinguole	96292.0	66.59	37.67	2.97	5.30	0.04	0.14	1286	12.33	N/A	http://tjj.xlgl.gov.cn/ywlm/tjgb/201603/t20160331_1587874.html
36	Huhehaote	101009.9	206.49	99.47	2.98	8.87	0.01	0.09	2011	6.57	17.32	http://www.tjcn.org/tjgb/05nmg/32831_2.html
37	Xingan	31391.0	73.94	85.97	4.93	12.75	0.07	0.15	1700	10.63	N/A	http://tjj.xam.gov.cn/xamtj/sjfbyjd/tjgb92/1373704/index.html
38	Chifeng	43269.0	202.32	227.63	6.95	29.36	0.03	0.13	4588	10.67	18.77	http://www.chifeng.gov.cn/contents/162/121119.html
39	Hulunbeier	63131.0	178.98	73.67	N/A	N/A	N/A	N/A	1953	7.73	21.93	http://www.tjcn.org/tjgb/05nmg/32623_3.html
40	Suzhou (Jiangsu)	136300.0	794.08	267.52	N/A	N/A	0.00	0.00	3102	2.92	14.99	http://tjj.suzhou.gov.cn/sztjj/tjgb/201702/67c94fa53b7a41f8b219e84bcf3b4c9f.shtml
41	Xuzhou	61511.0	573.02	293.88	2.73	15.57	0.00	0.05	4601	5.31	15.14	http://www.tjcn.org/tjgb/10js/32643_4.html
42	Taizhou (Jiangsu)	78756.0	285.69	178.47	N/A	N/A	0.05	0.05	1970	4.24	10.03	http://www.taizhou.gov.cn/art/2016/3/17/art_27_565202.html
43	Yancheng	58299.0	434.43	288.42	N/A	N/A	0.05	0.05	3242	4.49	12.41	http://www.tjcn.org/tjgb/10js/32648_3.html
44	Suqian	43853.0	269.53	215.85	N/A	N/A	N/A	N/A	2426	5.00	15.13	http://www.tjcn.org/tjgb/10js/32663_5.html
45	Yangzhou	89646.0	281.57	166.79	N/A	N/A	N/A	N/A	1980	4.42	18.35	http://www.yangzhou.gov.cn/xxgk_info/yz_xxgk/new_xxgk_descxxs.jsp?manuscriptid=2fb3dac0df0f400689c2dfce0affd74d
46	Lianyungang	48416.0	262.61	184.76	1.21	10.72	0.00	0.06	2708	6.05	14.40	http://www.lyg.gov.cn/zglygzfmhwz/tjgb/content/639b1472-f5b9-48e1-9cde-3fc7d89db33b.html

City ID	City	GDP per capita[1]	Population[2]		Population received *Dibao*[2]		Low-income population rate[3]		Health services		Green spaces[5]	Source
			Urban	Rural	Urban	Rural	Urban	Rural	Total	Access to health care[4]		
47	Chongqing	52330.0	1838.29	1178.26	37.52	50.26	0.02	0.04	19806	6.57	16.99	http://tjj.cq.gov.cn/zwgk_233/fdzdgknr/tjxx/sjzl_55471/tjgb_55472/202002/t20200219_5274452.html
48	Lanzhou	56972.0	298.96	70.35	6.28	9.44	0.02	0.13	2391	6.47	9.17	http://tjj.lanzhou.gov.cn/art/2016/3/21/art_4850_311660.html
49	Dingxi	10975.1	84.46	193.37	5.12	50.45	0.06	0.26	2775	9.99	16.39	http://www.dingxi.gov.cn/art/2018/10/2/art_423_364809.html
50	Pingliang	16595.0	76.09	133.71	5.25	21.73	0.07	0.16	2746	13.09	7.92	http://www.pingliang.gov.cn/zjpl/tjgb/201603/t20160325_89835.html
51	Zhangye	30704.0	51.46	70.52	4.83	8.34	0.09	0.12	1562	12.81	38.11	http://www.gs-zy.com/news/2016-03/29/content_2016080.htm
52	Jixi	28222.0	N/A	N/A	N/A	N/A	N/A	N/A	993	5.47	10.80	http://www.tjcn.org/tjgb/08hlj/32944_2.html
53	Qiqihaer	24436.0	339.80	209.60	10.09	14.74	0.03	0.07	967	1.76	10.02	http://www.tjcn.org/tjgb/08hlj/35550_4.html
54	Haerbin	59027.0	464.34	497.04	14.10	19.00	0.03	0.04	4070	4.23	9.53	http://www.harbin.gov.cn/art/2016/3/25/art_440_886001.html
55	Jiamusi	45575.0	N/A	N/A	N/A	N/A	N/A	N/A	752	3.14	14.15	http://www.tjcn.org/tjgb/08hlj/35247_3.html
56	Foshan	107715.7	705.46	37.60	0.71	1.90	0.00	0.05	1475	1.99	14.69	http://www.tjcn.org/tjgb/19gd/32758.html
57	Shenzhen	157985.0	1137.89	0.00	0.63	N/A	0.00	0.00	2946	2.59	16.91	http://sztqb.sznews.com/html/2016-04/24/content_3510204.htm
58	Chaozhou	33953.0	168.46	95.59	6.20	N/A	0.02	0.02	2333	8.84	10.57	http://www.tjcn.org/tjgb/19gd/32965_2.html
59	Guangzhou	134066.2	1154.75	195.36	2.40	3.30	0.00	0.02	3724	2.76	21.82	http://www.tjcn.org/tjgb/19gd/32754_6.html
60	Maoming	40324.0	243.35	364.73	19.12	N/A	0.03	0.03	3703	6.09	13.74	http://www.tjcn.org/tjgb/19gd/32761_3.html
61	Qingyuan	33400.0	188.20	195.25	0.80	10.10	0.00	0.05	2415	6.30	14.70	http://www.gdqy.gov.cn/xxgk/zzjg/zfjg/qystjj/tjxx/tjgb/content/post_67400.html
62	Jiangmen	49608.0	293.04	158.91	6.12	N/A	0.01	0.01	1681	3.72	17.62	http://www.jiangmen.gov.cn/home/tzgg/content/post_1783959.html
63	Benxi	67652.0	109.69	41.52	4.34	N/A	N/A	N/A	675	4.46	10.75	http://www.tjcn.org/tjgb/06ln/32894_4.html
64	Anshan	67675.0	179.20	166.80	3.88	3.72	0.02	0.02	1738	5.02	12.19	http://www.tjcn.org/tjgb/06ln/32845_3.html
65	Dalian	110673.0	547.30	151.40	4.60	6.30	0.01	0.04	2739	3.92	11.11	http://www.tjcn.org/tjgb/06ln/32844_7.html
66	Chaoyang	29120.0	N/A	N/A	6.30	8.10	N/A	N/A	4450	N/A	N/A	http://www.zgcy.gov.cn/ZGCY/zwgk/20160331/004034014_87685.htm
67	Jinzhou	44191.0	108.60	198.20	9.70	N/A	0.03	0.03	2446	7.97	13.50	http://www.tjcn.org/tjgb/06ln/32846_3.html
68	Yangquan	42688.0	92.09	47.74	3.85	3.99	0.04	0.08	1482	10.60	10.77	http://www.yq.gov.cn/ggsj_12505/tjgb/201712/t20171229_589069.shtml
69	Xinzhou	21731.0	145.50	168.60	4.78	14.28	0.03	0.08	5233	16.66	12.37	https://www.sxxz.gov.cn/ggsj/tjgb/201712/t20171218_111295.shtml
70	Yuncheng	22304.0	243.32	284.21	4.80	14.30	0.02	0.05	5410	10.26	10.63	https://www.yuncheng.gov.cn/doc/2019/10/29/2249.shtml
71	Linfen	26239.0	215.66	227.91	6.14	9.01	0.03	0.04	4389	9.89	10.53	http://www.linfen.gov.cn/contents/312/22965.html
72	Shanghai	103100.0	2115.78	299.49	17.57	3.09	0.01	0.01	5016	2.08	7.60	http://district.ce.cn/newarea/roll/201602/29/t20160229_9173984.shtml
73	Tianjin	106908.4	1278.40	268.55	13.07	10.37	0.01	0.04	5222	3.38	10.13	http://www.tj.gov.cn/sq/tjgb/202005/t20200520_2468074.html
74	Huzhou	70899.0	174.64	120.36	0.57	2.51	0.00	0.02	1377	4.67	16.58	http://www.tjcn.org/tjgb/11zj/32669_4.html
75	Ningbo	102475.0	556.36	226.14	4.90	N/A	0.01	0.01	4069	5.20	11.84	http://www.tjcn.org/tjgb/11zj/32666_4.html
76	Hangzhou	112268.0	679.06	222.74	1.39	7.43	0.00	0.03	4443	4.93	15.10	http://www.hangzhou.gov.cn/art/2016/3/24/art_805865_663727.html
77	Lishui	51632.0	120.64	93.26	0.71	6.81	0.01	0.07	1725	8.06	12.31	http://www.tjcn.org/tjgb/11zj/32674_3.html
78	Jiaxing	76834.0	279.23	179.27	2.13	N/A	0.00	0.00	1411	3.08	14.45	http://www.tjcn.org/tjgb/11zj/32668_3.html
79	Taizhou (Zhejiang)	59499.0	364.75	240.15	7.81	N/A	0.01	0.01	3455	5.71	12.78	http://www.zjtz.gov.cn/art/2019/1/15/art_1229049712_51830295.html
80	Siping	38687.7	119.60	206.80	17.63	N/A	0.05	0.05	2100	6.43	8.35	http://www.tjcn.org/tjgb/07jl/32849_3.html
81	Jilin	57506.1	223.05	217.50	10.60	11.49	0.05	0.05	3416	7.75	12.04	http://www.jl.gov.cn/sj/sjcx/ndbg/gdzs/201604/t20160420_66054

City ID	City	GDP per capita[1]	Population[2]		Population received *Dibao*[2]		Low-income population rate[3]		Health services		Green spaces[5]	Source
			Urban	Rural	Urban	Rural	Urban	Rural	Total	Access to health care[4]		
												30.html
82	Guilin	39329.0	231.29	264.87	4.97	25.86	0.02	0.10	5315	10.71	11.85	https://www.guilin.gov.cn/glsj/sjfb/tjgb/202005/t20200509_1774252.shtml
83	Hechi	17841.0	121.97	225.71	3.46	36.77	0.03	0.16	2287	6.58	N/A	http://www.tjcn.org/tjgb/20gx/32968_5.html
84	Nanning	49066.0	414.32	284.29	0.70	N/A	N/A	N/A	2759	3.95	12.77	http://tj.nanning.gov.cn/tjsj/tjgb/t295294.html
85	Yulin	25444.0	265.45	305.27	5.05	N/A	N/A	N/A	3490	6.12	9.96	http://www.tjcn.org/tjgb/20gx/32768_4.html
86	Bozhou	18771.0	186.74	317.96	2.40	18.40	0.01	0.06	1705	3.38	14.04	http://www.tjcn.org/tjgb/12ah/32683_5.html
87	Fuyang	16121.0	306.56	483.54	9.90	34.50	0.03	0.07	2644	3.35	12.31	http://tjj.fy.gov.cn/content/detail/5c37fd7c52550d9a67adaf01.html
88	Huainan	32298.0	165.20	74.50	4.98	4.48	0.03	0.06	1211	5.05	12.29	http://tjj.huainan.gov.cn/tjsj/tjgb/18956927.html
89	Anqing	29840.0	210.36	248.25	5.19	19.40	0.02	0.08	2497	5.44	13.25	http://aqxxgk.anqing.gov.cn/show.php?id=416312
90	Suzhou (Anhui)	22415.0	214.60	339.50	3.58	25.44	0.02	0.07	1880	3.39	12.89	http://www.tjcn.org/tjgb/12ah/32908_3.html
91	Luan	19885.0	245.90	331.60	5.03	N/A	N/A	N/A	2343	4.06	14.82	http://tjj.luan.gov.cn/sjla/tjgg/578141.html
92	Chaohu	31921.0	12.05	66.65	0.44	1.89	0.04	0.03	401	5.10	11.62	http://www.ahmhxc.com/tongjigongbao/4905.html
93	Xiangfan	60244.0	321.68	239.72	N/A	N/A	N/A	N/A	3713	6.61	12.65	http://www.xf.gov.cn/zxzx/jrgz/201604/t20160402_1175174.shtml
94	Enshi	20191.0	133.00	199.70	2.77	16.11	0.02	0.08	2990	8.99	N/A	http://www.enshi.gov.cn/sj/qztjgb/201604/t20160426_392311.shtml
95	Huanggang	25262.0	N/A	N/A	8.56	18.17	0.04	0.04	4244	6.75	14.15	http://www.hg.gov.cn/art/2016/3/15/art_13631_358396.html
96	Jingmen	47939.1	96.97	192.66	5.67	6.44	0.06	0.03	1986	6.86	11.62	http://www.tjcn.org/tjgb/17hb/32914_2.html
97	Weinan	27452.0	N/A	N/A	5.87	24.56	0.06	0.06	4246	7.92	12.28	http://www.weinan.gov.cn/gk/tjxx/tjgb/513054.htm
98	Baoji	47591.0	N/A	N/A	4.39	15.16	0.05	0.05	3011	8.00	12.28	http://tjj.baoji.gov.cn/art/2016/3/14/art_1959_182229.html
99	Yulin	77267.0	187.06	153.05	27.70	N/A	0.08	0.08	4861	14.29	18.67	http://www.yl.gov.cn/P/C/35664.htm
100	Hanzhong	30971.5	160.35	183.46	5.10	16.00	0.03	0.09	3814	11.09	13.20	http://www.hanzhong.gov.cn/hzszf/zwgk/tjxx/tjgb/201603/t20160329_320783.shtml
101	Binzhou	61189.0	212.75	175.11	N/A	N/A	N/A	N/A	2780	7.17	18.87	http://www.binzhou.gov.cn/zwgk/news/detail?code={81ae8270-c86e-4d27-ae76-2423c1e4d6df}
102	Qingdao	102519.0	636.66	273.04	N/A	N/A	N/A	N/A	3146	3.46	14.60	http://qdsq.qingdao.gov.cn/n15752132/n20546841/n32208957/n32209705/170630111459138305.html
103	Jinan	85919.0	484.69	228.51	2.20	8.10	0.00	0.04	5947	8.34	11.55	http://www.tjcn.org/tjgb/15sd/32860_6.html
104	Zaozhuang	52372.4	207.32	180.48	N/A	N/A	N/A	N/A	2457	6.34	14.60	http://www.tjcn.org/tjgb/15sd/32702_3.html
105	Linyi	36656.0	710.12	476.19	N/A	N/A	N/A	N/A	7002	5.90	19.07	http://tjj.linyi.gov.cn/info/1065/3662.htm
106	Liaocheng	31564.0	295.68	321.52	N/A	N/A	N/A	N/A	5619	9.10	12.63	http://www.tjcn.org/tjgb/15sd/32912.html
107	Weihai	106996.4	177.18	103.35	3.44	N/A	0.01	0.01	1144	4.08	25.05	http://www.weihai.gov.cn/art/2016/4/21/art_58862_1729512.html
108	Weifang	55824.0	517.67	410.05	1.73	14.72	0.00	0.04	7649	8.24	17.97	http://tjj.weifang.gov.cn/TJYW/TJFX/TJGB/201602/t20160225_1602541.htm
109	Dezhou	48062.0	297.05	277.18	2.30	15.90	0.01	0.06	4879	8.50	24.90	http://www.tjcn.org/tjgb/15sd/32712.html
110	Anyang	41700.8	209.72	191.28	5.42	20.59	0.03	0.11	5738	14.31	10.56	http://www.anyang.gov.cn/sitegroup/root/html/ff8080812b8fc534012bc280d47c5e84/20160422105523084.html
111	Luoyang	52036.2	355.02	319.28	6.72	22.62	0.02	0.07	895	1.33	9.23	http://www.ly.gov.cn/html/1/2/10/29/35/730268.html
112	Zhoukou	23644.0	333.43	547.49	59.00	N/A	0.07	0.07	7997	9.08	11.33	http://www.tjcn.org/tjgb/16hn/32725_3.html
113	Zhengzhou	77217.0	666.90	290.00	2.20	10.60	0.00	0.04	3923	4.10	7.10	http://tjj.zhengzhou.gov.cn/tjgb/3101703.jhtml
114	Puyang	36942.9	145.66	215.34	2.98	17.80	0.02	0.08	4137	11.46	14.29	http://www.pystj.gov.cn/show.asp?id=425
115	Pingdingshan	33104.0	206.14	198.06	5.71	15.02	0.03	0.08	3843	9.51	10.39	http://www.tjcn.org/tjgb/16hn/32863.html

City ID	City	GDP per capita[1]	Population[2]		Population received *Dibao*[2]		Low-income population rate[3]		Health services		Green spaces[5]	Source
			Urban	Rural	Urban	Rural	Urban	Rural	Total	Access to health care[4]		
116	Jiaozuo	55080.0	193.84	159.56	3.20	10.60	0.02	0.07	2810	7.95	11.07	http://www.tjcn.org/tjgb/16hn/32717_3.html
117	Xinyang	29321.0	273.73	366.27	12.35	36.09	0.05	0.10	4017	6.28	14.14	http://www.xytjj.gov.cn/www/tjzl/tjgb/2018/0329/25656.html
118	Shaoyang	19100.0	304.63	421.54	4.41	N/A	N/A	N/A	1137	1.57	10.97	http://tjj.hunan.gov.cn/tjfx/tjgb/szgb/sys/201603/t20160325_3798327.html
119	Yueyang	51273.4	304.02	258.90	4.25	N/A	N/A	N/A	4808	8.54	9.72	http://www.yueyang.gov.cn/tjgb/content_607892.html
120	Changsha	115443.0	552.78	190.40	19.87	N/A	0.03	0.03	4661	6.27	10.42	http://tjj.hunan.gov.cn/tjfx/tjgb/szgb/zss/201603/t20160314_4326869.html
121	Yiyang	30776.0	204.58	236.44	N/A	N/A	N/A	N/A	1096	2.49	9.00	http://www.yiyang.gov.cn/yiyang/2/80/86/content_217768.html
122	Loudi	33436.0	169.47	217.71	7.37	17.29	0.04	0.08	4014	10.37	9.55	http://www.tjcn.org/tjgb/18hn/32744_3.html
123	Changde	46408.0	278.10	306.30	12.21	25.07	0.04	0.08	5106	8.74	14.49	https://tjj.changde.gov.cn/zwgk/zfxxgk/tjxx1/tjgb/sj/content_637127
124	Qiannan	27888.0	N/A	N/A	2.93	24.49	0.08	0.08	N/A	0.00	N/A	http://www.tjcn.org/tjgb/24gz/32790.html
125	Qiandongnan	23311.0	N/A	N/A	4.46	62.43	0.19	0.19	3966	11.38	N/A	http://www.qdn.gov.cn/xxgk/zdgk/tjxx/tjnb/201607/t20160721_3757113.html

Note:

[1] CNY.

[2] per/10,000.

[3] %.

[4] /10 thousand [5] meter square per person
