# Supplementary material for: Long-term Care Insurance and Health and Perceived Satisfaction of Older Chinese: Comparisons Between Urban/Rural Areas, Chronic Conditions, and Their Intersectionality
Source: Int J Health Policy Manag. 2023 Dec 11;12:7938. doi: 10.34172/ijhpm.2023.7938 (PMC10843175; doi:10.34172/ijhpm.2023.7938)
Supplement: Supplementary file 2 — contains Tables S3-S11 and Figures S1-S2. [file ijhpm-12-7938-s002.pdf]

Article title: Long-term Care Insurance and Health and Perceived Satisfaction of Older Chinese: Comparisons Between Urban/Rural Areas, Chronic Conditions, and Their Intersectionality

Journal name: International Journal of Health Policy and Management (IJHPM)

Authors' information: Yinkai Zhang[1], Yu-Chih Chen[1,2]*, Julia Shu-Huah Wang[3]

[1]Department of Social Work & Social Administration, The University of Hong Kong, Hong Kong SAR, China.

[2]Social Policy Institute, Washington University in St. Louis, St. Louis, MO, USA.

[3]Department of Social Work, National Taiwan University, Taipei, Taiwan.

***Correspondence to:** Yu-Chih Chen; Email: yuchih@hku.hk

Citation: Zhang Y, Chen YC, Wang JSH. Long-term care insurance and health and perceived satisfaction of older Chinese: comparisons between urban/rural areas, chronic conditions, and their intersectionality. Int J Health Policy Manag. 2023;12:7938. doi:10.34172/ijhpm.2023.7938

Supplementary file 2

Table S3. Descriptive Statistics of Variables and Bivariate Analyses across Attrition Groups.

	Attrition		
Variables	Yes (*n*= 1,267)	No (*n*= 7,986)	t/χ^2
Age	70.08 (7.51)	67.73 (6.40)	t=–11.69***
Male	55.87%	48.64%	χ^2=22.78***
Above secondary school	9.88%	6.93%	χ^2=13.90***
Marital status			
Married	68.27%	77.75%	χ^2=54.55***
Partnered	5.21%	3.09%	χ^2=14.98***
Financial wealth	10.70 (2.45)	10.79 (2.03)	t=1.09
Baseline Self-rated health	2.02 (0.93)	2.10 (0.92)	t=2.94**
Baseline depressive symptoms	8.75 (6.77)	8.33 (6.50)	t=–2.06*
Baseline satisfaction with health services	3.34 (1.17)	3.38 (1.13)	t=–1.22

Note: Wealth was log-transformed. Means are out of the parentheses, standard deviations are in the parentheses.
*$p \leqslant .05$; **$p \leqslant .01$; ***$p \leqslant .001$

Table S4. The Comparison between Fixed and Random Effects (*N*=9,253).

	Self-rated health		Depressive symptoms		Satisfaction with health services	
Model	M3	M3b	M6	M6b	M9	M9b
	b	*b*	*b*	*b*	*b*	*b*
**Health care system**[1]						
Whether have LTCI policy	0.102***	0.103**	–0.154	–0.154	0.109*	0.109*
	(0.032)	(0.032)	(0.218)	(0.218)	(0.052)	(0.052)
**Environmental characteristics**[1]						
GDP	0.054	0.054	–0.377*	–0.377*	–0.071	–0.071
	(0.028)	(0.028)	(0.177)	(0.177)	(0.041)	(0.041)
Low-income population rate	–0.821**	–0.821**	5.547**	5.546**	–0.169	–0.169
	(0.278)	(0.278)	(1.746)	(1.745)	(0.352)	(0.352)
Urban	0.024	0.024	–0.133	–0.133	–0.040	–0.040
	(0.028)	(0.028)	(0.173)	(0.173)	(0.036)	(0.036)
Green spaces	–0.009***	–0.009***	0.044*	0.044*	0.005	0.005
	(0.003)	(0.003)	(0.019)	(0.019)	(0.005)	(0.005)
Number of health care clinics	–0.001	–0.001	0.017	0.017	0.012*	0.012*
	(0.004)	(0.004)	(0.026)	(0.026)	(0.006)	(0.006)
**Population characteristics**[2]						
Predisposing characteristics						
Age	–0.002	–0.002	–0.016	–0.016	0.005*	0.005*
	(0.002)	(0.002)	(0.013)	(0.013)	(0.003)	(0.003)
Male	0.014	0.014	–1.201***	–1.201***	–0.110**	–0.110**
	(0.032)	(0.032)	(0.213)	(0.213)	(0.038)	(0.038)
Above secondary school	0.003	0.003	–0.828**	–0.828**	0.002	0.002
	(0.043)	(0.043)	(0.273)	(0.273)	(0.053)	(0.053)
Urban *hukou*	0.034	0.034	–0.020	–0.020	–0.167***	–0.167***
	(0.030)	(0.030)	(0.204)	(0.204)	(0.040)	(0.040)
Marital status[3]						
Married	–0.033	–0.033	–0.117	–0.117	0.004	0.004
	(0.028)	(0.028)	(0.178)	(0.178)	(0.033)	(0.033)
Partnered	–0.018	–0.018	0.035	0.035	–0.007	–0.007
	(0.061)	(0.061)	(0.412)	(0.412)	(0.075)	(0.075)
Working	0.089***	0.089***	0.148	0.148	0.036	0.036
	(0.028)	(0.028)	(0.162)	(0.162)	(0.032)	(0.032)
Enabling resources						
Income	–0.002	–0.002	–0.032	–0.032	–0.001	–0.001
	(0.004)	(0.004)	(0.023)	(0.023)	(0.005)	(0.005)
Financial wealth	0.001	0.001	–0.134*	–0.134*	–0.008	–0.008
	(0.009)	(0.009)	(0.054)	(0.054)	(0.010)	(0.010)
Need						
Baseline chronic disease	–0.206***	–0.206***	0.575**	0.575**	–0.031	–0.031

Model	Self-rated health		Depressive symptoms		Satisfaction with health services	
	M3	M3b	M6	M6b	M9	M9b
	b	*b*	*b*	*b*	*b*	*b*
	(0.030)	(0.030)	(0.182)	(0.182)	(0.036)	(0.036)
Baseline self-rated health	0.353***	0.353***	–0.553***	–0.553***	0.093***	0.093***
	(0.013)	(0.013)	(0.082)	(0.082)	(0.015)	(0.015)
Baseline depressive symptoms	–0.019***	–0.019***	0.410***	0.410***	–0.009***	–0.009***
	(0.002)	(0.002)	(0.012)	(0.012)	(0.003)	(0.003)
Baseline satisfaction	0.042***	0.042***	–0.221***	–0.221***	0.258***	0.258***
	(0.009)	(0.009)	(0.059)	(0.059)	(0.012)	(0.012)
Baseline ADL	0.071***	0.071***	–0.226**	–0.226**	0.023	0.023
	(0.012)	(0.012)	(0.080)	(0.080)	(0.015)	(0.015)
Baseline IADL	0.032**	0.032**	–0.261**	–0.261**	–0.016	–0.016
	(0.013)	(0.013)	(0.086)	(0.086)	(0.015)	(0.015)
**Health behavior**[2]						
Personal health practices						
Drinking	0.057*	0.057*	–0.113	–0.113	0.015	0.015
	(0.024)	(0.024)	(0.147)	(0.147)	(0.029)	(0.029)
Smoking	–0.025	–0.025	0.148	0.148	–0.041	–0.041
	(0.029)	(0.029)	(0.183)	(0.183)	(0.038)	(0.038)
Physical activity	–0.032	–0.032	0.588*	0.588*	–0.065	–0.065
	(0.040)	(0.040)	(0.240)	(0.240)	(0.045)	(0.045)
Caregiving	0.039	0.039	–0.249	–0.249	0.028	0.028
	(0.031)	(0.031)	(0.195)	(0.195)	(0.040)	(0.040)
Social engagement	–0.008	–0.008	–0.192	–0.192	–0.037	–0.037
	(0.022)	(0.022)	(0.135)	(0.135)	(0.026)	(0.026)
Use of health services						
Medical expenditures	–0.014**	–0.014**	0.055	0.055	–0.017*	–0.017*
	(0.006)	(0.006)	(0.036)	(0.036)	(0.007)	(0.007)
Hospital utilization	–0.038	–0.038	0.050	0.050	–0.063	–0.063
	(0.024)	(0.024)	(0.166)	(0.166)	(0.032)	(0.032)
Attrition	0.007	0.007	–0.040	–0.040	0.022	0.022
	(0.037)	(0.037)	(0.222)	(0.222)	(0.038)	(0.038)
Random Effect						
Above secondary school		0.001		0.001		0.001
Income		0.001		0.001		0.001
Model statistics						
–2 Log Likelihood	23528.55	23528.35	57789.61	57782.35	27192.60	27200.80
AIC	23592.55	23593.55	57846.36	57843.96	27256.59	27265.03
BIC	23820.80	23826.08	58074.61	58063.64	27484.84	27494.12

Note: According to Chiavegatto Filho (2017), education and income varied across city levels, we allow education level and income as random slopes. Abbreviation: M, model. Standard errors are in the parentheses. M3, M6, and M9 (random intercept models) were extracted from Table 2, and M3a, M6a, and M9a were random

slope models. Results were combined using 20 imputed data sets. AIC = Akaike information criterion; BIC = Bayesian information criterion; Income, wealth, and GDP were log-transformed.
[1] City-level variables (n=125).
[2] Individual-level variables (n=9,253).
[3] Ref: not married and not partnered.
*$p \leqslant .05$; **$p \leqslant .01$; ***$p \leqslant .001$

Table S5. LTCI Pilots of the Study.

CityID	Cities	Whether surveyed in CHARLS	Whether included in this study period (2015-2018)
Implemented LTCI in 2016			
13	Chengdu	Yes	Yes
25	Chengde	Yes	Yes
29	Shangrao	Yes	Yes
40	Suzhou	Yes	Yes
47	Chongqing	Yes	Yes
53	Qiqihaer	Yes	Yes
59	Guangzhou	Yes	Yes
72	Shanghai	Yes	Yes
75	Ningbo	Yes	Yes
81	Jilin	Yes	Yes
89	Anqing	Yes	Yes
96	Jingmen	Yes	Yes
101	Binzhou	Yes	Yes
102	Qingdao	Yes	Yes
103	Jinan	Yes	Yes
104	Zaozhuang	Yes	Yes
105	Linyi	Yes	Yes
106	Liaocheng	Yes	Yes
107	Weihai	Yes	Yes
108	Weifang	Yes	Yes
109	Dezhou	Yes	Yes
N/A	Changchun	No	No
N/A	Dongying	No	No
N/A	Heze	No	No
N/A	Hunchun	No	No
N/A	Jining	No	No
N/A	Meihekou	No	No
N/A	Nantong	No	No
N/A	Rizhao	No	No
N/A	Shihezi	No	No
N/A	Songyuan	No	No
N/A	Taian	No	No
N/A	Tonghua	No	No
N/A	Yantai	No	No
N/A	Zibo	No	No
Implemented LTCI in 2020			
4	Kunming	Yes	No
9	Fuzhou	Yes	No
36	Huhehaote	Yes	No
73	Tianjin	Yes	No
84	Nanning	Yes	No
100	Hanzhong	Yes	No
N/A	Gannan	No	No
N/A	Jincheng	No	No
N/A	Kaifeng	No	No
N/A	Panjin	No	No
N/A	Qianxinan	No	No
N/A	Shijingshan District	No	No
N/A	Urumqi	No	No
N/A	Xiangtan	No	No
Total number of eligible pilots		21	

Note: Data source: http://www.gov.cn/zhengce/zhengceku/2020-11/05/5557630/files/ea1e0e04a6e349a9ae488622f69b3bc7.pdf

Table S6. Health conditions by the combination of locality and LTCI status (N=9,253).

	Urban with LTCI (n= 734)	Urban without LTCI (n= 2,920)	Rural with LTCI (n= 751)	Rural without LTCI (n= 4,848)	F
Baseline self-rated health	2.23 (0.92)	2.16 (0.90)	2.17 (1.00)	2.01 (0.90)	25.49***
Baseline depressive symptoms	6.89 (6.01)	7.24 (6.04)	8.50 (6.57)	9.27 (6.74)	75.65***
Baseline satisfaction	3.38 (1.08)	3.20 (1.09)	3.69 (1.09)	3.41 (1.15)	45.50***
Self-rated health	3.12 (1.02)	2.96 (0.97)	3.03 (1.08)	2.79 (0.99)	30.11***
Depressive symptoms	7.17 (6.34)	7.73 (6.26)	8.56 (6.39)	9.64 (6.75)	54.18***
Satisfaction with health services	3.35 (1.11)	3.26 (1.06)	3.64 (1.13)	3.41 (1.15)	22.77***

Note: Means are out of the parentheses, standard deviations are in the parentheses.
*p⩽.05; **p⩽.01; ***p⩽.001.

Table S7. LTCI status margins (N=9,253).

	Self-rated health	Depressive symptoms	Satisfaction with health services
**LTCI status*chronic conditions** (no LTCI vs. announced)			
No LTCI*without conditions	3.044***	8.308***	3.393***
	(0.028)	(0.176)	(0.037)
No LTCI*with conditions	2.837***	8.921***	3.363***
	(0.012)	(0.082)	(0.020)
Announced*without conditions	3.316***	9.025***	3.764***
	(0.151)	(1.042)	(0.196)
Announced*with conditions	3.119***	8.314***	3.634***
	(0.081)	(0.544)	(0.118)
**LTCI status*chronic conditions** (not implemented vs. implemented)			
Not implemented* without conditions	3.039***	8.315***	3.398***
	(0.029)	(0.185)	(0.039)
Not implemented* with conditions	2.834***	8.928***	3.359***
	(0.013)	(0.089)	(0.022)
Implemented*without conditions	3.120***	8.384***	3.421***
	(0.068)	(0.439)	(0.090)
Implemented*with conditions	2.903***	8.777***	3.437***
	(0.033)	(0.225)	(0.054)

Note. LTCI status margins are from Model 3a, 6a, and 9a. Margins are out of the parentheses, standard errors are in the parentheses. *p⩽.05; **p⩽.01; ***p⩽.001

Table S8. Effects of LTCI Policy on Health Outcomes, by Locality and Chronic Conditions (N=9,253).

	Locality		Chronic conditions	
	Urban (n= 3,654)	Rural (n= 5,599)	With 1+ chronic disease (n= 7,904)	Without chronic disease (n= 1,349)
Self-rated health	0.055	0.150***	0.091**	0.154
	(0.044)	(0.045)	(0.034)	(0.086)
Depressive symptoms	–0.063	–0.290	–0.247	0.444
	(0.283)	(0.310)	(0.242)	(0.458)
Satisfaction with health services	0.019	0.233**	0.111*	0.074
	(0.067)	(0.075)	(0.054)	(0.096)

Note: All models were adjusted for the city- and individual-level variables. Regression coefficients are out of the parentheses, standard errors are in the parentheses.
*p⩽.05; **p⩽.01; ***p⩽.001.

Table S9. Intersectionality analysis of LTCI Policy on Health Outcomes (N=9,253).

	Urban with 1+ chronic disease (n= 3,178)	Urban without chronic disease (n= 476)	Rural with 1+ chronic disease (n= 873)	Rural without chronic disease (n= 4,726)

Self-rated health	0.056	0.059	0.124**	0.267*
	(0.046)	(0.122)	(0.046)	(0.117)
Depressive symptoms	–0.161	0.431	–0.379	0.333
	(0.304)	(0.658)	(0.346)	(0.657)
Satisfaction with health services	0.026	–0.012	0.225**	0.146
	(0.074)	(0.134)	(0.076)	(0.141)

Note: All models were adjusted for the city- and individual-level variables. Regression coefficients are out of the parentheses, standard errors are in the parentheses.
**p*≤.05; ***p*≤.01; ****p*≤.001.

Table S10. Effects of LTCI Policy on Health Outcomes, by Age (*N*=18,645).

	Age	
	45-59 (*n*= 9,392)	60+ (*n*= 9,253)
Self-rated health	0.004	0.102***
	(0.034)	(0.032)
Depressive symptoms	–0.256	–0.154
	(0.233)	(0.218)
Satisfaction with health services	0.085*	0.109*
	(0.042)	(0.052)

Note: All models were adjusted for the city- and individual-level variables. Regression coefficients are out of the parentheses, standard errors are in the parentheses.
*p≤.05; **p≤.01; ***p≤.001.

Table S11. Effects of LTCI Policy on Health Outcomes, by Multimorbidity (*N*=9,253).

	Multimorbidity	
	With multimorbidity (*n*= 5,827)	Without multimorbidity (*n*= 3,426)
Self-rated health	0.083*	0.130**
	(0.038)	(0.051)
Depressive symptoms	–0.243	–0.046
	(0.264)	(0.309)
Satisfaction with health services	0.125*	0.071
	(0.059)	(0.070)

Note: All models were adjusted for the city- and individual-level variables. Regression coefficients are out of the parentheses, standard errors are in the parentheses.
*p≤.05; **p≤.01; ***p≤.001

Figure S1. The Comparison of LTCI Announcement and Implementation Effects on Health Outcomes (*N*=9,253).

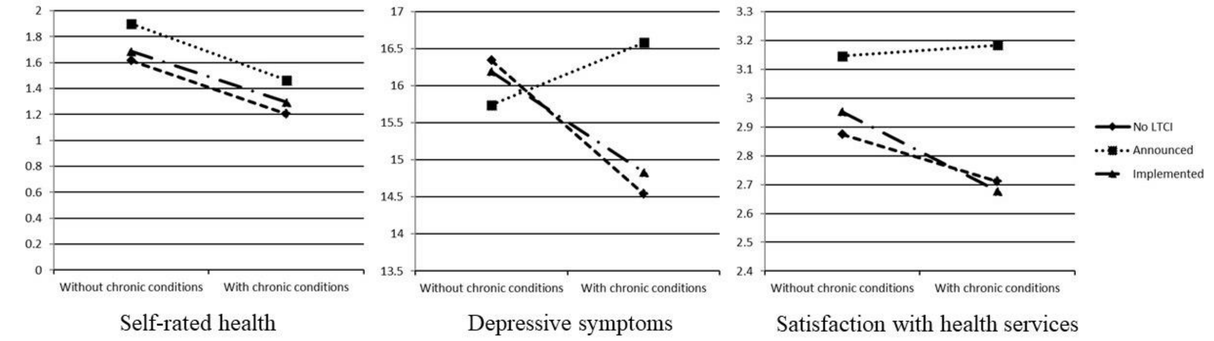

Note: All models were adjusted for the city- and individual-level variables. The value of the vertical coordinate shows the value of health outcomes.

Figure S2. The Comparison of LTCI Effects on Health Outcomes, Stratified by Locality and *Hukou* (*N*=9,253).

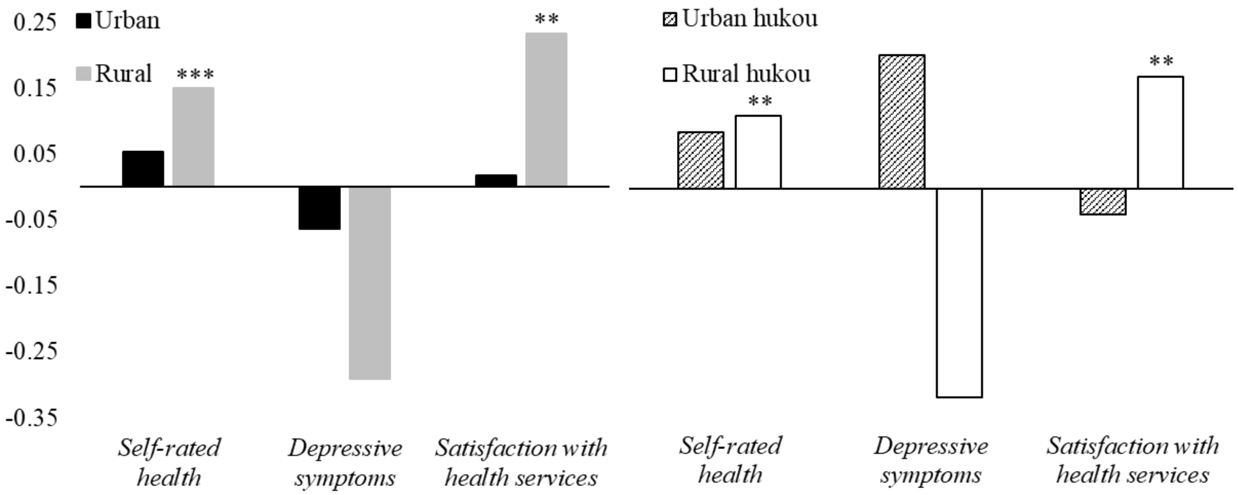

Note: All models were adjusted for the city- and individual-level variables. The value of the vertical coordinate shows the coefficient of policy impacts.
*$p \leqslant .05$; **$p \leqslant .01$; ***$p \leqslant .001$
